# Supplementary material for: Estimation of PM10 Levels and Sources in Air Quality Networks by Digital Analysis of Smartphone Camera Images Taken from Samples Deposited on Filters
Source: Sensors (Basel). 2019 Nov 4;19(21):4791. doi: 10.3390/s19214791 (PMC6864467; doi:10.3390/s19214791)
Supplement: Supplementary file 1 [file sensors-19-04791-s001.pdf]

Supplementary Material

Table S1. Values of RGB, HSV, HSL and Greyscale (Lu, Li, Avg) parameters obtained from image analysis of PM_{10} samples during 2015 in Badajoz (BA). Saharan dust outbreaks identified by official reports [27–29].

Date	Reference	PM_{10} (μg/m³)	Red (R)	Green (G)	Blue (B)	Hue (H_{HSV})	Saturation (S_{HSV})	Value (V)	Luminosity (Lu)	Lightness (Li)	Average (Avg)	Hue (H_{HSL})	Saturation (S_{HSL})	Luminance (L)
01/01/2015	1BA	14,76	126,00	117,67	89,33	46,33	29,00	49,00	117,43	107,67	111,00	46,37	17,03	42,22
02/01/2015	2BA	17,68	104,67	98,00	73,67	47,00	29,67	41,00	97,70	89,17	92,11	47,11	17,38	34,97
03/01/2015	3BA	22,71	71,67	69,33	50,67	53,33	29,67	28,00	68,52	61,17	63,89	53,37	17,17	23,99
04/01/2015	4BA	19,46	92,33	87,00	66,00	47,67	28,33	36,00	86,65	79,17	81,78	47,86	16,63	31,05
05/01/2015	5QBA	21,90	86,67	83,33	62,67	51,67	27,67	34,00	82,59	74,67	77,56	51,81	16,06	29,28
06/01/2015	6BA	11,39	138,67	133,33	113,67	47,33	18,00	54,33	133,08	126,17	128,56	47,20	9,91	49,48
07/01/2015	7BA	10,13	155,67	150,33	131,00	47,00	15,33	61,33	150,10	143,33	145,67	47,00	11,04	56,21
08/01/2015	8BA	16,42	119,67	113,33	93,00	46,00	22,33	47,00	113,24	106,33	108,67	45,73	12,54	41,70
09/01/2015	9BA	22,13	108,67	102,33	82,33	45,33	24,33	42,67	102,26	95,50	97,78	45,58	13,79	37,45
10/01/2015	10QBA	25,40	88,33	84,00	61,33	50,33	30,67	34,67	83,32	74,83	77,89	50,42	18,04	29,35
11/01/2015	11BA	20,28	99,33	93,00	71,67	46,00	27,67	39,00	92,84	85,50	88,00	46,24	16,18	33,53
12/01/2015	12BA	33,63	63,00	64,67	51,33	67,67	20,67	25,00	63,38	58,00	59,67	67,47	11,49	22,75
13/01/2015	13BA	29,12	86,33	81,00	62,67	46,67	27,67	34,00	80,84	74,50	76,67	46,49	15,88	29,22
14/01/2015	14BA	25,03	86,67	82,00	65,00	47,00	24,67	34,00	81,79	75,83	77,89	47,10	14,28	29,74
15/01/2015	15QBA	16,90	153,33	146,67	119,33	48,00	22,33	60,00	146,15	136,33	139,78	48,23	14,33	53,46
16/01/2015	16QhBA	10,90	180,33	180,67	168,33	61,67	7,00	71,00	179,73	174,50	176,44	61,54	7,66	68,43
17/01/2015	17BA	8,32	154,67	149,67	130,67	48,00	15,67	60,33	149,39	142,67	145,00	47,50	10,68	55,95
18/01/2015	18BA	2,45	189,00	188,33	183,00	53,33	3,00	74,33	188,10	186,00	186,78	53,33	4,35	72,94
19/01/2015	19BA	4,85	186,33	183,67	174,33	46,67	6,00	73,00	183,57	180,33	181,44	46,67	8,04	70,72
20/01/2015	20BA	8,06	148,67	144,33	124,33	49,33	16,33	58,33	143,84	136,50	139,11	49,33	10,27	53,53
21/01/2015	21QBA	8,10	169,00	166,33	149,33	52,00	11,67	66,33	165,70	159,17	161,56	51,84	10,26	62,42
22/01/2015	22BA	5,40	186,00	183,00	174,00	45,00	6,33	73,00	183,00	180,00	181,00	44,93	8,00	70,59
23/01/2015	23BA	4,07	165,67	162,00	149,67	46,33	10,00	65,00	161,91	157,67	159,11	46,25	8,22	61,83
24/01/2015	24BA	8,45	128,67	123,33	102,33	47,67	20,33	50,67	122,98	115,50	118,11	47,83	11,40	45,29
25/01/2015	25BA	9,44	142,67	136,67	110,33	49,00	22,67	56,00	136,08	126,50	129,89	48,86	12,78	49,61

26/01/2015	26QBA	12,30	110,00	104,00	83,00	46,67	24,67	43,33	103,79	96,50	99,00	46,68	13,99	37,84
27/01/2015	27BA	16,03	101,33	94,33	75,00	44,00	26,00	40,00	94,45	88,17	90,22	44,05	14,94	34,58
04/02/2015	28BA	5,09	180,67	179,00	170,00	50,67	6,00	71,00	178,72	175,33	176,56	50,81	6,69	68,76
05/02/2015	29BA	6,35	177,67	176,00	164,00	52,33	7,67	69,67	175,51	170,83	172,56	52,75	8,12	66,99
06/02/2015	30BA	8,42	151,33	147,33	128,67	49,67	14,67	59,67	146,87	140,00	142,44	49,41	9,85	54,90
07/02/2015	31QBA	6,80	176,67	173,67	158,00	50,33	10,33	69,33	173,20	167,33	169,44	50,33	10,65	65,62
08/02/2015	32QhBA	8,20	179,00	176,00	160,33	50,67	10,67	70,00	175,53	169,67	171,78	50,35	10,94	66,54
09/02/2015	33BA	17,10	114,33	108,67	86,33	48,00	24,33	45,00	108,29	100,33	103,11	47,85	13,96	39,35
10/02/2015	34BA	18,70	117,67	111,67	90,33	47,00	23,33	46,33	111,43	104,00	106,56	46,83	13,14	40,78
11/02/2015	35BA	24,46	126,33	120,67	97,00	48,67	23,33	49,33	120,20	111,67	114,67	48,44	13,14	43,79
12/02/2015	36BA	24,48	132,67	126,00	101,67	46,67	23,00	52,00	125,70	117,17	120,11	47,10	13,23	45,95
13/02/2015	37QBA	15,70	149,33	144,67	117,33	51,33	21,67	58,33	143,73	133,33	137,11	51,25	13,15	52,29
14/02/2015	38BA	11,37	187,00	185,00	179,00	45,00	4,33	73,00	185,00	183,00	183,67	44,84	5,55	71,76
15/02/2015	39BA	7,83	199,33	196,67	193,00	36,00	3,33	78,33	196,97	196,17	196,33	36,00	5,39	76,93
16/02/2015	40BA	6,56	176,33	173,67	164,33	46,67	7,00	69,00	173,57	170,33	171,44	46,67	7,09	66,80
17/02/2015	41BA	4,88	193,67	192,33	187,67	47,00	3,33	75,67	192,29	190,67	191,22	46,95	4,66	74,77
18/02/2015	42QBA	5,30	182,33	183,00	168,33	62,67	8,00	71,67	181,83	175,67	177,89	62,67	9,25	68,89
19/02/2015	43BA	11,38	149,33	146,00	129,00	50,33	13,67	58,67	145,51	139,17	141,44	50,19	8,78	54,58
20/02/2015	44BA	17,02	134,33	130,00	109,00	49,33	19,00	53,00	129,44	121,67	124,44	49,75	10,41	47,71
21/02/2015	45BA	7,48	171,33	169,67	157,67	52,33	8,00	67,00	169,18	164,50	166,22	52,75	7,55	64,51
22/02/2015	46BA	7,96	177,67	175,00	165,00	47,33	7,00	69,33	174,86	171,33	172,56	47,31	7,57	67,19
23/02/2015	48QhBA	7,10	194,33	194,33	183,00	60,00	6,00	76,00	193,54	188,67	190,56	59,87	8,81	74,05
24/02/2015	49QBA	10,80	190,33	190,33	178,67	60,00	6,00	75,00	189,52	184,50	186,44	60,00	8,27	72,35
25/02/2015	50QBA	10,20	174,67	173,00	157,67	54,00	10,00	68,67	172,28	166,17	168,44	54,12	9,57	65,16
26/02/2015	51QBA	9,40	160,00	156,67	137,33	51,00	14,00	62,67	156,01	148,67	151,33	51,15	10,66	58,30
27/02/2015	52QBA	7,80	189,00	188,00	173,67	56,33	8,00	74,67	187,21	181,33	183,56	56,33	10,66	71,18
28/02/2015	53QBA	6,20	190,33	190,33	178,33	60,00	6,00	75,00	189,49	184,33	186,33	60,00	8,49	72,29
01/03/2015	54QBA	6,90	192,00	192,00	180,33	60,00	6,00	75,33	191,18	186,17	188,11	60,00	8,47	73,01
02/03/2015	55QBA	6,30	175,33	174,67	159,67	58,00	9,00	69,00	173,76	167,50	169,89	57,92	9,17	65,75
03/03/2015	56QBA	9,10	148,33	145,33	125,00	52,33	15,67	58,33	144,54	136,67	139,56	52,25	9,86	53,59
04/03/2015	57QBA	7,20	168,00	165,33	148,00	52,00	12,00	65,67	164,68	158,00	160,44	52,00	10,31	61,96
05/03/2015	58QBA	12,20	162,33	158,33	133,00	51,67	18,00	63,67	157,40	147,67	151,22	51,80	13,66	57,91

06/03/2015	59QBA	18,40	130,33	122,00	90,67	47,67	30,33	51,00	121,56	110,50	114,33	47,40	17,95	43,33
07/03/2015	60QBA	16,30	136,67	129,67	99,00	48,67	27,67	53,67	128,99	117,83	121,78	48,88	15,99	46,21
08/03/2015	61QBA	12,30	145,67	140,33	114,67	50,00	21,33	57,33	139,66	130,17	133,56	49,68	12,41	51,05
09/03/2015	62BA	12,98	128,33	124,00	105,00	49,33	18,33	50,67	123,58	116,67	119,11	48,88	10,00	45,75
10/03/2015	63QBA	16,10	128,33	123,33	98,67	50,00	23,00	50,33	122,66	113,50	116,78	49,83	13,07	44,51
11/03/2015	64QBA	15,60	142,00	137,33	110,00	51,33	22,33	55,67	136,40	126,00	129,78	51,28	12,70	49,41
12/03/2015	65BA	55,12	147,00	139,00	102,00	49,33	30,67	57,67	138,09	124,50	129,33	49,33	18,07	48,82
13/03/2015	66QBA	56,30	151,67	140,33	94,00	48,00	38,33	59,67	139,47	122,83	128,67	48,21	23,47	48,17
14/03/2015	67QBA	10,50	176,33	173,33	156,33	51,00	11,00	69,00	172,77	166,33	168,67	51,00	11,28	65,23
15/03/2015	68BA	7,57	162,67	159,33	146,33	47,67	10,00	64,00	159,12	154,50	156,11	47,72	8,13	60,59
16/03/2015	69QBA	14,90	155,00	151,33	128,67	51,67	17,00	60,67	150,52	141,83	145,00	51,77	11,64	55,62
17/03/2015	70QBA	14,20	162,67	161,00	142,67	54,67	12,00	64,00	160,07	152,67	155,44	54,78	9,77	59,87
18/03/2015	71BA	4,19	180,00	180,00	172,67	60,00	4,00	71,00	179,49	176,33	177,56	60,00	4,66	69,15
19/03/2015	72QBA	7,70	177,33	175,67	161,00	54,00	9,00	69,33	174,99	169,17	171,33	54,08	9,51	66,34
20/03/2015	73QBA	17,40	167,33	163,00	142,00	49,67	15,33	65,33	162,44	154,67	157,44	49,81	12,62	60,65
21/03/2015	74BA	9,47	159,00	157,00	144,33	51,67	9,00	62,33	156,53	151,67	153,44	51,81	7,10	59,48
22/03/2015	75QBA	6,70	184,33	183,67	171,33	57,00	7,33	72,33	182,94	177,83	179,78	57,03	8,43	69,74
23/03/2015	76QBA	8,60	178,33	177,00	161,67	55,33	9,33	70,00	176,21	170,00	172,33	55,42	9,81	66,67
24/03/2015	77BA	3,57	184,67	183,67	177,67	51,33	3,67	72,33	183,46	181,17	182,00	51,31	4,74	71,05
25/03/2015	78QBA	6,20	192,67	193,00	180,67	61,67	6,67	75,67	192,07	186,83	188,78	61,54	9,04	73,27
26/03/2015	79QBA	10,00	183,33	182,33	168,67	56,33	8,00	72,00	181,59	176,00	178,11	56,22	9,27	69,02
27/03/2015	80BA	6,31	179,00	177,33	170,00	49,67	5,00	70,33	177,17	174,50	175,44	49,56	5,59	68,43
28/03/2015	81QBA	10,10	169,67	167,00	151,33	51,33	11,00	66,67	166,46	160,50	162,67	51,31	9,70	62,94
29/03/2015	82QBA	7,50	183,00	180,33	165,33	51,00	9,67	71,67	179,84	174,17	176,22	50,98	10,93	68,30
30/03/2015	83BA	7,31	170,00	169,00	157,67	55,00	7,33	67,00	168,42	163,83	165,56	55,00	6,76	64,25
31/03/2015	84BA	11,65	151,33	148,33	130,67	51,00	13,67	59,33	147,73	141,00	143,44	51,29	9,06	55,29
01/04/2015	85QBA	14,10	158,67	154,33	133,00	50,00	16,33	62,33	153,75	145,83	148,67	49,90	11,75	57,19
02/04/2015	86QBA	12,40	172,33	169,00	151,67	50,33	12,00	67,67	168,49	162,00	164,33	50,33	11,12	63,53
03/04/2015	87BA	10,25	167,67	165,00	152,33	49,67	9,33	65,67	164,67	160,00	161,67	49,67	8,07	62,75
04/04/2015	88QBA	11,70	154,33	150,33	130,00	50,33	15,67	60,67	149,75	142,17	144,89	50,26	10,79	55,75
05/04/2015	89QBA	19,30	160,00	156,00	132,33	51,00	17,33	62,67	155,18	146,17	149,44	51,32	12,71	57,32
06/04/2015	90BA	11,54	155,67	153,67	138,33	53,00	11,00	61,00	153,01	147,00	149,22	53,19	8,03	57,65

07/04/2015	91QBA	13,60	167,67	165,33	149,33	52,67	10,67	65,67	164,70	158,50	160,78	52,39	9,48	62,16
08/04/2015	92QBA	14,60	172,33	171,00	155,67	55,67	9,67	67,67	170,21	164,00	166,33	55,56	9,37	64,38
09/04/2015	93BA	11,36	164,67	112,67	150,00	51,67	8,67	64,67	126,20	138,67	142,44	136,45	33,41	52,75
10/04/2015	94QBA	13,40	159,33	157,67	142,00	55,00	11,00	63,00	156,92	150,67	153,00	54,76	8,49	59,15
11/04/2015	95QBA	6,60	179,00	179,00	164,67	60,00	8,33	70,33	178,00	171,83	174,22	59,84	8,81	67,45
12/04/2015	96BA	17,76	168,67	166,33	151,33	52,00	10,67	66,33	165,77	160,00	162,11	51,94	9,12	62,75
13/04/2015	97QBA	31,90	160,00	154,67	126,33	50,33	20,67	62,67	153,80	143,17	147,00	50,50	15,05	56,14
14/04/2015	98QBA	44,80	159,00	150,67	118,33	47,67	25,67	62,33	150,15	138,67	142,67	47,71	17,48	54,38
15/04/2015	99BA	17,85	157,00	155,00	141,67	52,33	10,00	61,67	154,49	149,33	151,22	52,17	7,26	58,56
16/04/2015	100QBA	11,40	182,67	181,33	166,33	55,33	9,00	72,00	180,56	174,50	176,78	55,37	10,37	68,50
17/04/2015	101QBA	18,00	178,67	176,00	159,67	51,67	10,67	70,33	175,42	169,17	171,44	51,65	11,07	66,34
18/04/2015	102BA	9,11	190,00	189,00	183,00	51,33	4,00	74,67	188,79	186,50	187,33	51,43	5,11	73,14
19/04/2015	103QBA	6,80	195,67	195,33	185,00	58,33	5,67	76,67	194,68	190,33	192,00	58,18	8,25	74,64
20/04/2015	104QBA	9,00	172,00	171,67	157,33	58,67	8,33	67,33	170,73	164,67	167,00	58,75	8,11	64,58
21/04/2015	105BA	14,72	154,33	151,67	136,00	51,33	12,00	60,33	151,13	145,17	147,33	51,31	8,35	56,93
22/04/2015	106QBA	19,50	182,33	179,00	164,33	49,00	10,00	71,33	178,67	173,33	175,22	48,89	11,02	67,97
23/04/2015	107QBA	15,50	170,33	166,67	147,33	50,67	13,33	66,67	166,08	158,83	161,44	50,57	11,97	62,29
24/04/2015	108BA	15,32	166,67	164,00	146,67	52,00	12,00	65,33	163,35	156,67	159,11	52,00	10,17	61,44
25/04/2015	109QBA	10,10	193,33	193,33	183,33	60,00	5,00	76,00	192,63	188,33	190,00	60,00	7,50	73,86
26/04/2015	110QBA	6,10	204,00	204,00	193,33	60,00	5,33	80,00	203,25	198,67	200,44	60,00	9,45	77,91
27/04/2015	111BA	7,20	191,67	189,67	184,67	43,00	3,67	75,33	189,74	188,17	188,67	43,10	5,25	73,79
28/04/2015	112QBA	8,20	192,33	191,33	181,33	55,00	6,00	75,33	190,84	186,83	188,33	54,55	8,07	73,27
29/04/2015	113QBA	8,30	196,00	196,00	185,67	60,00	5,67	77,00	195,28	190,83	192,56	60,00	8,33	74,90
30/04/2015	114BA	8,61	178,67	176,00	168,33	44,33	6,00	70,00	176,02	173,50	174,33	44,36	6,34	68,04
01/05/2015	115QBA	9,80	159,33	159,00	145,00	58,67	9,00	62,33	158,09	152,17	154,44	58,75	6,97	59,67
02/05/2015	116QBA	10,80	170,00	169,67	154,67	58,67	9,00	66,67	168,69	162,33	164,78	58,83	8,47	63,73
03/05/2015	117BA	11,86	174,67	171,67	162,67	45,00	7,00	68,67	171,67	168,67	169,67	45,00	6,95	66,14
04/05/2015	118QBA	17,90	184,00	181,00	164,67	51,00	10,33	72,33	180,49	174,33	176,56	50,68	11,98	68,37
05/05/2015	119QBA	12,70	192,67	192,67	182,33	60,00	5,33	75,67	191,94	187,50	189,22	60,00	7,65	73,53
06/05/2015	120BA	8,84	168,67	166,33	157,00	48,33	7,00	66,00	166,17	162,83	164,00	48,18	6,33	63,86
07/05/2015	121QBA	13,60	174,33	173,67	160,00	58,33	8,67	68,67	172,85	167,17	169,33	58,17	8,57	65,69
08/05/2015	122QBA	14,60	192,00	192,00	182,00	60,00	5,00	75,00	191,30	187,00	188,67	60,00	7,35	73,33

09/05/2015	123BA	11,35	168,67	166,00	156,00	47,33	7,67	66,00	165,86	162,33	163,56	47,31	6,83	63,66
10/05/2015	124QBA	18,20	169,00	163,67	144,33	47,33	14,67	66,33	163,43	156,67	159,00	47,03	12,55	61,44
11/05/2015	125QBA	21,50	167,67	163,00	139,67	50,00	16,33	66,00	162,35	153,67	156,78	49,97	13,82	60,26
12/05/2015	126BA	27,87	123,67	118,00	96,00	47,67	22,67	48,67	117,65	109,83	112,56	47,70	12,60	43,07
13/05/2015	127QBA	34,00	147,67	140,33	111,67	47,67	24,33	57,67	139,87	129,67	133,22	47,79	14,44	50,85
14/05/2015	128QBA	14,80	184,67	182,00	167,00	51,00	9,67	72,67	181,51	175,83	177,89	50,98	11,16	68,95
15/05/2015	129BA	11,13	186,67	184,67	177,33	48,00	5,00	73,33	184,57	182,00	182,89	47,60	6,40	71,37
16/05/2015	130QBA	8,80	191,33	190,67	178,67	56,67	6,67	75,00	189,97	185,00	186,89	56,92	9,05	72,55
17/05/2015	131QBA	11,90	177,67	175,00	159,67	51,00	10,00	69,67	174,49	168,67	170,78	51,11	10,43	66,14
18/05/2015	132BA	18,99	158,33	155,00	136,00	51,33	14,00	62,00	154,37	147,17	149,78	51,07	10,36	57,71
19/05/2015	133QBA	15,90	188,33	186,00	172,33	51,00	8,33	74,00	185,53	180,33	182,22	51,26	10,70	70,72
20/05/2015	134QBA	11,70	189,67	189,33	178,67	58,33	5,67	74,67	188,66	184,17	185,89	58,33	7,76	72,22
21/05/2015	135BA	10,22	187,00	184,00	175,00	45,00	6,00	73,33	184,00	181,00	182,00	45,00	8,11	70,98
22/05/2015	136QBA	13,90	193,33	190,33	175,33	50,00	9,00	75,67	189,91	184,33	186,33	50,00	12,74	72,29
23/05/2015	137QBA	13,80	186,33	183,33	168,67	49,67	9,67	73,00	182,94	177,50	179,44	49,80	11,40	69,61
24/05/2015	138BA	13,61	176,33	174,00	162,00	50,00	8,33	69,00	173,65	169,17	170,78	50,19	8,35	66,34
25/05/2015	139QBA	17,30	170,33	167,33	151,67	50,67	11,00	66,67	166,87	161,00	163,11	50,35	9,93	63,14
26/05/2015	140QBA	13,00	192,00	189,33	174,00	51,33	9,33	75,33	188,82	183,00	185,11	51,16	12,50	71,76
27/05/2015	141BA	16,49	157,67	154,67	139,33	50,33	11,33	62,00	154,22	148,50	150,56	50,18	8,61	58,24
28/05/2015	142QBA	23,00	154,33	150,00	125,33	51,00	18,67	60,33	149,18	139,83	143,22	50,96	12,58	54,84
29/05/2015	143QBA	22,90	171,33	166,33	146,67	48,00	14,67	67,00	166,01	159,00	161,44	47,83	12,85	62,35
30/05/2015	144BA	18,44	173,67	171,67	158,33	52,33	8,67	68,00	171,15	166,00	167,89	52,14	8,61	65,10
31/05/2015	145QBA	11,50	193,33	193,33	181,00	60,00	6,33	76,00	192,47	187,17	189,22	60,00	9,09	73,40
01/06/2015	146QBA	19,40	187,33	184,33	168,00	50,67	10,00	73,67	183,82	177,67	179,89	50,65	12,49	69,67
02/06/2015	147BA	14,95	173,67	171,33	159,33	50,33	8,33	68,33	170,98	166,50	168,11	50,38	8,10	65,29
03/06/2015	148QBA	22,40	169,33	166,33	149,33	51,33	11,67	66,33	165,77	159,33	161,67	50,96	10,44	62,48
04/06/2015	149QBA	27,10	158,00	153,67	135,00	48,67	14,33	61,67	153,27	146,50	148,89	48,70	10,60	57,45
05/06/2015	150BA	25,17	146,33	141,67	120,00	49,00	18,33	57,33	141,13	133,17	136,00	49,34	10,81	52,22
06/06/2015	151QBA	51,60	153,67	145,33	111,33	48,33	27,33	60,00	144,70	132,50	136,78	48,19	17,28	51,96
07/06/2015	152QBA	45,80	169,33	160,33	130,67	46,33	23,00	66,67	160,15	150,00	153,44	46,03	18,41	58,82
08/06/2015	153BA	32,20	155,00	149,67	122,67	50,33	21,00	61,00	148,90	138,83	142,44	50,09	13,92	54,44
09/06/2015	154QBA	29,10	177,00	171,67	151,67	47,33	14,33	69,33	171,39	164,33	166,78	47,38	13,97	64,44

10/06/2015	155QBA	32,10	177,00	171,67	152,00	47,33	14,00	69,33	171,41	164,50	166,89	47,20	13,81	64,51
11/06/2015	156BA	24,84	177,00	173,33	160,67	46,67	9,33	69,33	173,22	168,83	170,33	46,54	9,48	66,21
12/06/2015	157QBA	15,70	189,00	189,00	178,67	60,00	5,33	74,33	188,28	183,83	185,56	60,00	7,26	72,09
13/06/2015	158QBA	15,00	190,67	190,67	180,67	60,00	5,00	75,00	189,97	185,67	187,33	60,00	7,21	72,81
14/06/2015	159BA	7,84	190,00	188,33	182,33	47,33	4,00	74,67	188,26	186,17	186,89	47,14	5,57	73,01
15/06/2015	160QBA	9,80	189,67	189,33	179,33	58,33	5,33	74,33	188,70	184,50	186,11	58,18	7,33	72,35
16/06/2015	161QBA	10,10	180,33	180,67	167,00	61,33	7,67	71,00	179,64	173,83	176,00	61,43	8,42	68,17
17/06/2015	162BA	10,72	172,00	169,00	159,67	45,33	7,33	67,33	168,98	165,83	166,89	45,38	6,92	65,03
18/06/2015	163QBA	11,00	172,33	171,33	156,67	56,33	9,00	67,33	170,52	164,50	166,78	56,40	8,65	64,51
19/06/2015	164QBA	16,70	157,67	154,33	136,00	50,67	14,00	62,00	153,75	146,83	149,33	50,75	10,01	57,58
20/06/2015	165BA	18,00	135,00	130,33	110,33	48,67	18,67	53,00	129,91	122,67	125,22	48,63	10,06	48,10
21/06/2015	166QBA	18,80	165,67	161,67	142,67	50,00	13,67	65,00	161,18	154,17	156,67	49,63	11,41	60,46
22/06/2015	167QBA	19,10	180,00	177,00	161,67	50,33	10,33	71,00	176,56	170,83	172,89	50,18	10,89	66,99
23/06/2015	168BA	13,41	182,67	180,67	173,00	48,00	5,33	71,33	180,55	177,83	178,78	47,88	6,26	69,74
24/06/2015	169QBA	11,20	180,67	180,33	169,00	58,33	6,67	71,00	179,61	174,83	176,67	58,18	7,27	68,56
25/06/2015	170QBA	15,30	163,00	160,33	142,67	52,00	12,67	64,00	159,66	152,83	155,33	51,99	9,94	59,93
26/06/2015	171BA	19,14	150,00	145,67	125,67	49,33	16,33	58,67	145,18	137,83	140,44	49,30	10,38	54,05
27/06/2015	172QBA	17,50	155,00	151,00	133,67	49,00	14,00	60,67	150,63	144,33	146,56	48,97	9,64	56,60
28/06/2015	173QBA	16,00	164,00	161,00	143,67	51,00	12,33	64,00	160,42	153,83	156,22	51,14	10,05	60,33
29/06/2015	174BA	22,11	159,67	155,33	135,33	49,33	15,33	62,33	154,84	147,50	150,11	49,33	11,32	57,84
30/06/2015	175QBA	31,20	160,00	155,00	135,33	48,00	15,67	62,67	154,67	147,67	150,11	47,83	11,49	57,91
01/07/2015	176QBA	14,50	184,33	184,33	174,33	60,00	5,00	72,33	183,63	179,33	181,00	60,00	6,61	70,33
02/07/2015	177BA	7,81	192,67	190,33	185,00	42,33	4,00	75,67	190,45	188,83	189,33	42,14	5,80	74,05
03/07/2015	178QBA	12,10	176,67	176,67	164,67	60,00	7,00	69,00	175,83	170,67	172,67	60,00	7,11	66,93
04/07/2015	179QBA	10,80	182,67	182,00	169,67	57,00	7,33	71,67	181,28	176,17	178,11	57,14	8,48	69,15
05/07/2015	180BA	11,10	186,67	184,67	176,33	48,67	5,33	73,33	184,50	181,50	182,56	48,78	7,04	71,18
06/07/2015	181QBA	17,20	177,00	174,33	157,00	52,00	11,33	69,33	173,68	167,00	169,44	52,04	11,36	65,49
07/07/2015	182QBA	23,40	129,67	120,33	92,33	45,00	29,00	51,00	120,33	111,00	114,11	44,98	16,82	43,53
08/07/2015	183BA	22,60	116,67	110,00	87,33	46,67	25,33	46,67	109,81	102,00	104,67	46,37	14,38	40,00
09/07/2015	184QBA	22,70	166,33	163,33	145,33	51,33	12,67	65,33	162,70	155,83	158,33	51,39	10,59	61,11
10/07/2015	185QBA	20,40	186,33	186,33	175,00	60,00	5,67	73,00	185,54	180,67	182,56	60,00	7,62	70,85
11/07/2015	186BA	14,72	169,67	168,67	156,00	55,33	8,00	67,00	167,99	162,83	164,78	55,71	7,61	63,92

12/07/2015	187QBA	14,30	184,33	182,00	167,00	51,67	9,33	72,33	181,44	175,67	177,78	51,90	10,92	68,89
13/07/2015	188QBA	18,60	171,67	168,00	151,33	49,67	11,67	67,33	167,60	161,50	163,67	49,32	10,88	63,33
14/07/2015	189BA	22,71	161,67	158,33	143,33	49,33	11,33	63,67	157,98	152,50	154,44	48,98	8,94	59,80
15/07/2015	190QBA	24,00	163,00	158,67	131,00	52,00	19,67	64,33	157,64	147,00	150,89	51,88	14,82	57,65
16/07/2015	191QBA	17,90	175,33	172,67	152,33	53,33	13,33	68,67	171,80	163,83	166,78	53,27	12,61	64,25
17/07/2015	192BA	21,15	151,00	145,00	126,33	45,67	16,67	59,00	144,95	138,67	140,78	45,40	10,60	54,38
18/07/2015	193QBA	9,30	190,33	190,67	180,33	61,67	5,33	75,00	189,87	185,50	187,11	61,82	7,44	72,75
19/07/2015	194QBA	8,60	193,00	193,33	183,00	62,00	5,33	75,67	192,54	188,17	189,78	62,00	7,73	73,79
20/07/2015	195BA	11,31	183,00	180,67	173,33	45,67	5,00	71,67	180,64	178,17	179,00	45,78	6,29	69,87
21/07/2015	196QBA	11,80	191,00	191,00	181,00	60,00	5,00	75,00	190,30	186,00	187,67	60,00	7,25	72,94
22/07/2015	197QBA	12,60	186,00	186,00	174,67	60,00	6,33	73,00	185,21	180,33	182,22	59,72	7,83	70,78
23/07/2015	198BA	17,07	163,67	160,33	145,00	49,33	11,67	64,33	159,96	154,33	156,33	49,30	9,27	60,52
24/07/2015	199QBA	8,50	196,67	196,33	186,00	58,33	5,67	77,00	195,68	191,33	193,00	58,18	8,38	75,03
25/07/2015	200QBA	13,20	188,00	185,33	174,00	48,67	7,33	74,00	185,10	181,00	182,44	48,84	9,46	70,98
26/07/2015	201BA	15,36	176,00	173,67	162,33	49,33	7,67	69,00	173,36	169,17	170,67	49,67	7,96	66,34
27/07/2015	202QBA	9,80	195,00	194,33	185,00	56,33	5,33	76,33	193,82	190,00	191,44	56,36	7,70	74,51
28/07/2015	203QBA	13,40	183,33	182,00	168,33	54,67	8,00	72,00	181,32	175,83	177,89	54,78	9,48	68,95
29/07/2015	204BA	10,69	182,67	179,67	172,33	42,67	5,33	71,33	179,78	177,50	178,22	42,55	6,67	69,61
30/07/2015	205QBA	7,50	201,33	201,00	191,33	58,00	5,00	79,00	200,39	196,33	197,89	58,00	8,52	76,99
31/07/2015	206QBA	8,10	196,00	196,00	186,00	60,00	5,00	77,00	195,30	191,00	192,67	60,00	7,81	74,90
07/08/2015	213BA	23,56	103,00	97,33	73,00	48,67	29,00	40,33	96,82	88,00	91,11	48,67	17,05	34,51
08/08/2015	214QBA	23,40	132,67	126,33	105,67	45,67	20,67	52,00	126,22	119,17	121,56	45,97	11,33	46,73
10/08/2015	216BA	45,26	141,33	133,00	98,00	48,67	30,67	55,67	132,30	119,67	124,11	48,47	18,11	46,93
11/08/2015	217QBA	22,70	168,00	164,67	145,67	51,33	13,33	65,67	164,04	156,83	159,44	51,18	11,39	61,50
12/08/2015	218QBA	14,50	184,33	181,67	169,67	49,33	8,00	72,33	181,39	177,00	178,56	49,46	9,40	69,41
13/08/2015	219BA	7,40	194,00	193,00	189,00	49,33	2,67	76,00	192,93	191,50	192,00	49,33	3,94	75,10
14/08/2015	220QBA	7,60	195,33	195,33	185,33	60,00	5,00	76,33	194,63	190,33	192,00	60,00	7,73	74,64
15/08/2015	221QBA	6,40	200,67	200,33	190,67	58,00	5,00	78,33	199,73	195,67	197,22	58,00	8,43	76,73
16/08/2015	222BA	5,35	197,67	196,33	193,67	40,00	2,33	77,67	196,43	195,67	195,89	40,00	3,36	76,73
17/08/2015	223QBA	7,90	199,00	199,00	189,00	60,00	5,00	78,00	198,30	194,00	195,67	60,00	8,20	76,08
18/08/2015	224QBA	14,30	179,33	179,33	169,00	60,00	6,00	70,33	178,61	174,17	175,89	60,00	6,39	68,30
19/08/2015	225BA	20,11	159,33	156,33	140,33	50,67	12,00	62,33	155,84	149,83	152,00	50,51	9,03	58,76

20/08/2015	226QBA	27,90	152,33	147,33	122,00	50,33	19,67	59,67	146,61	137,17	140,56	50,18	12,88	53,79
21/08/2015	227QBA	25,10	180,67	175,67	156,67	47,67	13,33	71,00	175,39	168,67	171,00	47,52	13,90	66,14
22/08/2015	228BA	9,25	185,33	184,00	176,33	51,00	5,00	73,00	183,74	180,83	181,89	51,11	6,07	70,92
23/08/2015	229QBA	11,60	195,33	195,67	185,00	61,67	5,67	76,67	194,85	190,33	192,00	61,82	8,25	74,64
24/08/2015	230QBA	14,10	199,67	199,00	189,00	56,67	5,33	78,33	198,44	194,33	195,89	56,36	8,79	76,21
25/08/2015	231BA	12,77	159,67	157,67	145,67	51,00	9,00	62,67	157,25	152,67	154,33	51,43	6,84	59,87
26/08/2015	232QBA	11,20	182,33	182,33	172,00	60,00	5,67	71,33	181,61	177,17	178,89	60,00	6,64	69,48
27/08/2015	233QBA	10,90	191,00	190,67	179,33	58,33	6,00	75,00	189,94	185,17	187,00	58,33	8,35	72,61
28/08/2015	234BA	12,55	166,33	165,67	155,67	56,67	6,67	65,00	165,11	161,00	162,56	56,36	5,67	63,14
29/08/2015	235QBA	36,10	156,67	147,00	103,67	49,33	33,67	61,67	146,00	130,17	135,78	49,08	21,23	51,05
31/08/2015	237BA	15,25	175,33	173,00	161,33	49,67	8,00	69,00	172,67	168,33	169,89	50,00	8,08	66,01
01/09/2015	238QBA	15,80	181,00	177,67	165,00	47,67	9,00	70,67	177,48	173,00	174,56	47,50	9,76	67,84
02/09/2015	239QBA	15,90	192,33	188,33	176,67	44,67	8,00	75,67	188,36	184,50	185,78	44,67	11,12	72,35
03/09/2015	240BA	16,09	176,33	174,33	166,33	48,33	5,67	69,00	174,19	171,33	172,33	48,32	5,98	67,19
04/09/2015	241QBA	19,10	182,67	178,33	165,00	45,00	9,67	71,67	178,31	173,83	175,33	45,23	10,89	68,17
05/09/2015	242QBA	20,40	169,33	166,33	149,33	51,00	12,00	66,33	165,77	159,33	161,67	50,94	10,44	62,48
06/09/2015	243BA	18,34	148,00	144,33	125,33	50,33	15,33	58,00	143,77	136,67	139,22	50,37	9,58	53,59
07/09/2015	244QBA	24,10	141,00	135,67	109,33	50,00	22,33	55,33	134,94	125,17	128,67	49,88	12,79	49,08
08/09/2015	245QBA	23,30	162,00	155,00	135,33	44,33	16,33	63,67	155,09	148,67	150,78	44,29	12,55	58,30
09/09/2015	246BA	17,61	160,67	157,67	143,00	50,00	11,00	63,00	157,27	151,83	153,78	49,76	8,56	59,54
10/09/2015	247QBA	11,50	193,33	190,67	179,33	48,67	7,33	76,00	190,43	186,33	187,78	48,64	10,19	73,07
11/09/2015	248QBA	15,80	177,00	173,33	160,33	46,67	9,33	69,33	173,19	168,67	170,22	46,67	9,66	66,14
12/09/2015	249BA	10,91	189,00	188,33	180,00	55,33	5,00	74,33	187,89	184,50	185,78	55,56	6,38	72,35
13/09/2015	250QBA	12,00	207,67	204,67	196,33	44,00	5,67	81,67	204,71	202,00	202,89	44,00	10,68	79,22
14/09/2015	251QBA	12,20	189,67	188,00	180,00	50,33	5,00	74,33	187,79	184,83	185,89	50,36	6,88	72,48
15/09/2015	252BA	16,50	188,67	186,00	178,33	44,67	5,33	74,00	186,02	183,50	184,33	44,55	7,23	71,96
16/09/2015	253QBA	10,40	202,00	199,00	190,67	44,00	5,67	79,33	199,05	196,33	197,22	44,00	9,66	76,99
17/09/2015	254QBA	9,50	189,67	189,00	178,67	57,00	5,67	74,67	188,42	184,17	185,78	57,14	7,75	72,22
18/09/2015	255BA	8,65	156,00	154,33	142,00	52,67	9,00	61,33	153,82	149,00	150,78	52,86	6,60	58,43
19/09/2015	256QBA	12,00	152,67	147,00	122,67	48,67	19,67	60,00	146,49	137,67	140,78	48,67	12,80	53,99
20/09/2015	257QBA	17,40	144,33	138,00	113,00	47,67	21,67	56,67	137,58	128,67	131,78	47,89	12,80	50,46
21/09/2015	258BA	17,70	132,33	126,00	101,67	47,33	23,00	52,00	125,63	117,00	120,00	47,59	13,11	45,88

22/09/2015	259QBA	12,60	181,00	178,00	161,33	51,00	11,00	71,00	177,46	171,17	173,44	50,78	11,72	67,12
23/09/2015	260QBA	18,70	158,33	152,00	127,33	47,67	19,33	62,00	151,60	142,83	145,89	47,73	13,83	56,01
24/09/2015	261BA	25,10	129,00	123,00	95,00	49,00	26,33	50,67	122,30	112,00	115,67	49,41	15,18	43,92
25/09/2015	262QBA	23,40	161,33	155,67	135,33	47,00	16,33	63,33	155,43	148,33	150,78	46,91	12,18	58,17
26/09/2015	263QBA	20,60	161,00	156,00	135,00	48,33	16,00	63,33	155,58	148,00	150,67	48,45	12,15	58,04
27/09/2015	264BA	19,88	142,00	137,00	116,33	48,33	18,00	55,67	136,60	129,17	131,78	48,30	10,20	50,65
28/09/2015	265QBA	19,30	133,67	125,67	103,33	44,33	22,67	52,33	125,78	118,50	120,89	44,16	12,81	46,47
29/09/2015	266QBA	14,10	164,00	159,33	139,33	48,67	15,00	64,33	158,91	151,67	154,22	48,67	11,94	59,48
30/09/2015	267BA	12,52	147,00	144,00	128,00	50,67	13,00	57,67	143,51	137,50	139,67	50,51	8,09	53,92
01/10/2015	268QBA	33,20	154,67	148,67	129,00	46,00	16,33	60,67	148,55	141,83	144,11	46,00	11,35	55,62
02/10/2015	269QBA	22,10	150,67	142,33	113,00	47,00	25,00	59,00	142,03	131,83	135,33	46,74	15,29	51,70
03/10/2015	270BA	20,78	151,00	145,33	117,33	49,67	22,33	59,00	144,56	134,17	137,89	49,87	13,93	52,61
04/10/2015	271QBA	32,20	177,33	170,33	147,00	46,33	17,00	69,33	170,17	162,17	164,89	46,14	16,34	63,59
05/10/2015	272QBA	15,50	202,67	199,67	189,67	46,00	6,33	79,67	199,60	196,17	197,33	45,99	11,06	76,93
06/10/2015	273BA	11,82	187,67	187,33	181,67	56,67	3,00	73,33	187,01	184,67	185,56	56,67	4,27	72,42
07/10/2015	274QBA	11,20	169,00	165,67	151,33	48,67	10,67	66,33	165,36	160,17	162,00	48,63	9,32	62,81
08/10/2015	275QBA	11,60	162,33	156,67	140,67	44,67	13,33	63,67	156,74	151,50	153,22	44,33	10,47	59,41
09/10/2015	276BA	19,21	131,67	125,33	100,67	47,33	23,33	51,67	124,94	116,17	119,22	47,74	13,34	45,56
10/10/2015	277QBA	14,80	156,00	153,00	132,00	52,67	15,67	61,33	152,16	144,00	147,00	52,59	10,81	56,47
11/10/2015	278QBA	6,10	194,00	192,00	183,33	49,67	5,67	76,00	191,81	188,67	189,78	49,55	8,04	73,99
12/10/2015	279BA	4,09	186,00	185,33	179,33	53,33	3,33	73,00	185,05	182,67	183,56	53,33	4,61	71,63
13/10/2015	280QBA	9,10	164,33	162,33	150,33	51,00	8,67	64,33	161,91	157,33	159,00	51,43	7,17	61,70
14/10/2015	281QBA	8,60	168,00	166,00	154,00	51,00	8,00	65,67	165,58	161,00	162,67	51,43	7,45	63,14
15/10/2015	282BA	13,70	116,33	111,67	91,67	48,67	21,33	45,33	111,25	104,00	106,56	48,67	11,86	40,78
16/10/2015	283QBA	28,00	124,67	114,67	83,67	45,67	33,33	48,67	114,60	104,17	107,67	45,38	19,67	40,85
17/10/2015	284QBA	15,60	158,00	153,00	134,00	48,00	15,00	62,00	152,72	146,00	148,33	47,50	11,02	57,25
18/10/2015	285BA	4,67	183,00	180,67	174,33	43,67	4,33	71,67	180,71	178,67	179,33	43,50	5,67	70,07
19/10/2015	286QBA	11,40	186,67	185,67	177,67	54,00	4,67	73,33	185,32	182,17	183,33	54,00	6,18	71,44
20/10/2015	287QBA	7,90	179,67	178,00	169,00	50,67	6,33	70,67	177,72	174,33	175,56	50,67	6,61	68,37
21/10/2015	288BA	11,25	151,33	148,33	130,33	51,33	13,67	59,33	147,70	140,83	143,33	51,42	9,20	55,23
22/10/2015	289QBA	19,40	130,33	124,67	102,33	47,67	21,33	51,00	124,29	116,33	119,11	47,86	12,05	45,62
23/10/2015	290QBA	22,70	104,00	98,00	80,00	45,33	23,00	40,67	98,00	92,00	94,00	45,00	13,06	36,08

24/10/2015	291BA	14,96	118,67	114,33	94,67	49,33	20,00	46,67	113,87	106,67	109,22	49,19	11,25	41,83
25/10/2015	292QBA	14,40	154,00	150,00	134,00	48,33	13,00	60,33	149,72	144,00	146,00	48,21	9,00	56,47
26/10/2015	293QBA	8,50	178,00	176,00	164,00	51,00	8,00	69,67	175,58	171,00	172,67	51,43	8,33	67,06
27/10/2015	294BA	5,13	187,33	187,00	181,33	56,67	3,33	73,33	186,67	184,33	185,22	56,67	4,24	72,29
28/10/2015	295QBA	8,60	190,33	190,33	181,33	60,00	4,67	74,67	189,70	185,83	187,33	60,00	6,50	72,88
29/10/2015	296QBA	12,60	117,33	111,33	95,33	44,00	19,00	46,00	111,47	106,33	108,00	43,64	10,35	41,70
30/10/2015	297BA	12,91	131,00	126,33	108,00	48,00	18,00	51,00	126,03	119,50	121,78	47,83	9,62	46,86
31/10/2015	298QBA	6,70	181,33	181,33	172,00	60,00	5,33	71,00	180,68	176,67	178,22	60,00	5,96	69,28
01/11/2015	299QBA	32,20	187,33	186,00	175,00	50,67	6,67	73,67	185,51	181,17	182,78	53,72	8,36	71,05
02/11/2015	300BA	4,76	195,67	195,00	190,00	53,33	3,00	76,67	194,79	192,83	193,56	53,33	4,56	75,62
03/11/2015	301QBA	47,40	173,00	168,67	153,67	46,67	11,33	68,00	168,53	163,33	165,11	46,63	10,55	64,05
04/11/2015	302QBA	32,80	183,67	179,33	168,33	43,00	8,33	72,33	179,47	176,00	177,11	43,08	9,70	69,02
05/11/2015	303BA	15,90	147,00	143,33	123,67	50,67	16,00	57,67	142,73	135,33	138,00	50,58	9,75	53,07
06/11/2015	304QBA	12,10	148,00	146,00	125,33	55,00	15,33	58,00	144,97	136,67	139,78	54,70	9,58	53,59
07/11/2015	305QBA	26,30	142,00	137,33	115,33	49,33	18,67	55,67	136,77	128,67	131,56	49,51	10,81	50,46
08/11/2015	306BA	27,04	143,67	140,33	123,33	50,33	14,33	56,00	139,84	133,50	135,78	50,18	8,37	52,35
09/11/2015	307QBA	30,50	134,00	127,00	105,33	45,00	21,33	52,67	126,95	119,67	122,11	45,30	11,98	46,93
10/11/2015	308QBA	34,40	137,67	131,67	109,33	47,33	20,67	54,00	131,36	123,50	126,22	47,27	11,47	48,43
11/11/2015	309BA	36,69	106,67	101,67	78,00	49,67	26,67	42,00	101,06	92,33	95,44	49,53	15,52	36,21
12/11/2015	310QBA	66,90	119,00	113,33	93,33	47,00	21,33	46,67	113,12	106,17	108,56	46,70	12,11	41,63
13/11/2015	311QBA	41,30	118,67	114,00	95,33	48,00	19,67	46,33	113,67	107,00	109,33	47,93	10,91	41,96
14/11/2015	312BA	38,84	108,67	105,00	87,00	50,00	19,67	42,67	104,51	97,83	100,22	49,83	11,07	38,37
15/11/2015	313QBA	28,00	126,67	120,33	102,33	44,33	19,33	49,67	120,40	114,50	116,44	44,17	10,68	44,90
16/11/2015	314QBA	23,30	109,00	104,33	91,33	44,33	16,33	42,67	104,40	100,17	101,56	44,24	8,82	39,28
17/11/2015	315BA	32,99	102,00	99,00	82,67	51,00	19,00	40,00	98,49	92,33	94,56	50,75	10,47	36,21
18/11/2015	316QBA	27,30	111,67	106,67	90,33	46,33	19,33	44,00	106,57	101,00	102,89	45,97	10,56	39,61
19/11/2015	317QBA	31,60	111,67	105,67	88,33	44,67	21,00	43,67	105,71	100,00	101,89	44,55	11,67	39,22
20/11/2015	318BA	27,12	82,00	80,00	64,33	53,00	21,67	32,00	79,32	73,17	75,44	53,20	12,07	28,69
21/11/2015	319QBA	29,90	182,33	175,33	156,33	44,00	14,00	71,67	175,47	169,33	171,33	43,85	15,18	66,41
22/11/2015	320QBA	5,90	194,33	194,00	186,00	57,67	4,33	76,00	193,51	190,17	191,44	57,78	6,42	74,58
23/11/2015	321BA	4,38	177,33	175,00	165,33	48,33	7,00	69,67	174,81	171,33	172,56	48,33	7,17	67,19
24/11/2015	322QBA	13,20	160,33	155,67	138,33	47,67	13,67	63,00	155,43	149,33	151,44	47,46	10,42	58,56

25/11/2015	323QBA	15,60	150,67	144,33	125,00	45,33	17,00	59,00	144,31	137,83	140,00	45,23	10,95	54,05
26/11/2015	324BA	14,34	123,67	118,00	96,33	47,33	22,33	48,67	117,67	110,00	112,67	47,58	12,42	43,14
27/11/2015	325QBA	19,10	116,67	111,67	95,00	46,33	18,33	45,67	111,55	105,83	107,78	46,20	10,23	41,50
28/11/2015	326QBA	26,30	129,33	123,67	105,00	46,33	18,67	50,33	123,55	117,17	119,33	46,03	10,39	45,95
29/11/2015	327BA	8,95	146,67	143,33	127,33	50,00	13,33	57,67	142,91	137,00	139,11	49,68	8,19	53,73
30/11/2015	328QBA	24,90	136,00	128,67	109,67	43,33	19,33	53,33	128,88	122,83	124,78	43,30	10,72	48,17
01/12/2015	329QBA	26,10	82,00	77,00	60,33	46,33	26,67	32,00	76,88	71,17	73,11	46,15	15,28	27,91
02/12/2015	330BA	22,96	83,67	80,33	64,00	50,00	23,67	32,67	79,89	73,83	76,00	49,97	13,32	28,95
03/12/2015	331QBA	20,20	128,33	122,33	96,00	48,67	25,33	50,33	121,75	112,17	115,56	48,90	14,41	43,99
04/12/2015	332QBA	23,50	114,67	108,33	87,00	46,00	24,33	45,00	108,17	100,83	103,33	46,24	13,72	39,54
05/12/2015	333BA	22,29	81,00	79,00	63,67	53,00	21,33	31,67	78,35	72,33	74,56	53,14	11,98	28,37
06/12/2015	334QBA	28,60	88,67	83,33	63,67	47,33	28,00	35,00	83,08	76,17	78,56	47,20	16,41	29,87
07/12/2015	335QBA	28,00	121,00	112,33	87,00	44,67	28,00	47,33	112,38	104,00	106,78	44,71	16,35	40,78
08/12/2015	336BA	16,13	129,67	124,67	105,33	48,00	18,67	51,00	124,36	117,50	119,89	47,67	10,35	46,08
09/12/2015	337QBA	24,30	123,67	116,00	92,33	45,33	25,33	48,33	115,95	108,00	110,67	45,32	14,50	42,35
10/12/2015	338QBA	20,40	141,33	135,33	113,00	47,67	20,00	55,33	135,03	127,17	129,89	47,33	11,33	49,87
11/12/2015	339BA	17,46	121,00	116,33	96,33	48,67	20,67	47,00	115,91	108,67	111,22	48,67	11,35	42,61
12/12/2015	340QBA	25,90	108,67	102,67	79,33	48,00	27,00	42,33	102,29	94,00	96,89	47,72	15,60	36,86
13/12/2015	341QBA	26,30	124,00	116,33	91,00	46,00	26,33	48,33	116,17	107,50	110,44	46,09	15,35	42,16
14/12/2015	342BA	28,88	106,67	101,67	82,00	48,00	23,33	42,00	101,34	94,33	96,78	47,83	13,08	36,99
15/12/2015	343QBA	41,70	88,67	82,00	59,33	46,67	33,00	35,00	81,81	74,00	76,67	46,42	19,83	29,02
16/12/2015	344QBA	44,10	127,00	114,33	80,33	44,00	37,00	49,67	114,61	103,67	107,22	43,75	22,50	40,65
17/12/2015	345BA	38,56	68,67	68,67	50,33	60,00	27,00	27,00	67,38	59,50	62,56	60,00	15,64	23,40
18/12/2015	346QBA	35,50	81,67	74,67	54,67	44,33	33,00	32,00	74,74	68,17	70,33	44,43	19,80	26,73
19/12/2015	347QBA	43,70	79,67	71,67	46,00	46,00	42,33	31,33	71,55	62,83	65,78	45,79	26,78	24,64
20/12/2015	348BA	16,41	107,33	102,33	83,00	48,00	22,33	42,00	102,03	95,17	97,56	47,67	12,78	37,32
21/12/2015	349QBA	8,80	166,67	164,33	151,67	50,67	9,00	65,33	163,94	159,17	160,89	50,67	7,83	62,42
22/12/2015	350QBA	8,20	166,67	163,67	150,00	49,00	10,00	65,33	163,34	158,33	160,11	49,19	8,62	62,09
23/12/2015	351BA	8,41	148,33	146,00	133,67	50,33	10,00	58,00	145,63	141,00	142,67	50,48	6,43	55,29
24/12/2015	352QBA	13,36	141,33	138,67	124,33	50,33	12,00	55,33	138,22	132,83	134,78	50,59	6,96	52,09
25/12/2015	353QBA	14,50	145,67	140,67	121,00	48,00	17,00	57,00	140,34	133,33	135,78	47,82	10,16	52,29
26/12/2015	354BA	20,67	101,33	97,33	79,33	49,33	22,00	39,67	96,91	90,33	92,67	49,17	12,18	35,42

27/12/2015	355QBA	21,20	118,67	112,33	90,00	46,67	24,33	46,67	112,10	104,33	107,00	46,71	13,75	40,92
28/12/2015	356QBA	19,50	162,00	157,00	136,33	48,00	16,00	63,67	156,60	149,17	151,78	48,31	12,13	58,50
29/12/2015	357BA	7,24	140,00	136,67	123,67	47,67	11,67	55,00	136,46	131,83	133,44	47,76	6,63	51,70
30/12/2015	358QBA	12,40	140,33	134,67	114,67	47,00	18,67	55,33	134,46	127,50	129,89	46,85	10,15	50,00
31/12/2015	359QBA	6,30	176,67	173,00	159,67	47,00	9,67	69,00	172,84	168,17	169,78	46,96	9,79	65,95

Saharan Dust Outbreaks days are identified in orange color.

Table S2. Values of RGB, HSV, HSL and Greyscale (Lu, Li, Avg) parameters obtained from image analysis of PM_{10} samples during 2015 in Monfragüe (MF). Saharan dust outbreaks identified by official reports [27–29].

Date	Reference	PM_{10} (µg/m³)	Red (R)	Green (G)	Blue (B)	Hue (H_{HSV})	Saturation (S_{HSV})	Value (V)	Luminosity (Lu)	Lightness (Li)	Average (Avg)	Hue (H_{HSL})	Saturation (S_{HSL})	Luminance (L)
01/01/2015	1096MF	9,80	156,67	152,33	127,67	51,00	18,33	61,67	151,52	142,17	145,56	51,00	12,85	55,75
02/01/2015	1097MF	13,70	154,00	149,67	127,33	50,33	17,33	60,33	149,01	140,67	143,67	50,32	11,66	55,16
03/01/2015	1098MF	17,60	117,67	111,00	84,00	48,33	29,00	46,00	110,51	100,83	104,22	48,13	16,69	39,54
04/01/2015	1099MF	20,30	97,33	91,00	71,00	45,33	27,00	38,33	90,93	84,17	86,44	45,58	15,64	33,01
05/01/2015	1100QMF	22,30	102,00	95,33	74,00	45,67	27,67	40,33	95,24	88,00	90,44	45,71	15,91	34,51
06/01/2015	1101MF	62,70	127,67	122,00	100,00	47,67	21,67	50,33	121,65	113,83	116,56	47,70	12,15	44,64
07/01/2015	1102MF	18,00	139,67	134,67	114,33	48,00	18,00	55,00	134,29	127,00	129,56	48,15	10,00	49,80
08/01/2015	1103MF	22,10	112,00	107,33	87,33	48,67	22,00	44,00	106,91	99,67	102,22	48,63	12,37	39,08
09/01/2015	1104MF	19,00	102,33	97,33	78,00	48,00	23,67	40,00	97,03	90,17	92,56	47,67	13,49	35,36
10/01/2015	1105QMF	18,60	123,67	118,00	96,33	47,33	22,00	48,67	117,67	110,00	112,67	47,58	12,43	43,14
11/01/2015	1106MF	15,00	118,00	112,67	89,00	49,00	24,67	46,00	112,13	103,50	106,56	48,98	14,01	40,59
12/01/2015	1107MF	14,50	130,33	125,00	100,00	49,33	23,33	51,00	124,37	115,17	118,44	49,44	13,17	45,16
13/01/2015	1108MF	10,50	152,33	147,00	126,67	47,33	16,67	60,00	146,70	139,50	142,00	47,54	11,11	54,71
14/01/2015	1109MF	15,00	150,00	146,00	125,00	50,33	17,00	58,67	145,37	137,50	140,33	50,40	10,64	53,92
15/01/2015	1110QMF	1,80	174,00	169,00	148,67	48,00	14,33	68,00	168,63	161,33	163,89	48,15	13,52	63,27
16/01/2015	1111MF	4,50	206,33	204,67	195,67	51,00	5,00	80,67	204,39	201,00	202,22	50,73	9,87	78,82
17/01/2015	1112MF	6,60	188,67	185,67	171,67	49,33	9,00	74,33	185,32	180,17	182,00	49,39	11,36	70,65
18/01/2015	1113MF	2,60	197,00	194,33	185,33	46,33	6,00	77,33	194,26	191,17	192,22	46,36	9,14	74,97

19/01/2015	1114MF	3,10	204,67	202,67	193,33	49,67	5,33	80,00	202,43	199,00	200,22	49,55	10,12	78,04
20/01/2015	1115QMF	3,70	191,67	186,33	171,00	44,67	10,67	75,33	186,38	181,33	183,00	44,55	14,02	71,11
21/01/2015	1116MF	3,70	190,33	188,33	177,33	51,00	6,67	74,67	187,98	183,83	185,33	51,04	9,14	72,09
22/01/2015	1117MF	4,40	196,33	194,00	184,33	48,33	6,00	77,00	193,81	190,33	191,56	48,33	9,28	74,64
23/01/2015	1118MF	4,70	192,67	191,33	179,67	53,67	6,67	75,67	190,80	186,17	187,89	53,94	9,44	73,01
24/01/2015	1119MF	5,60	189,33	186,33	173,67	48,67	8,00	74,33	186,08	181,50	183,11	48,50	10,66	71,18
25/01/2015	1120QMF	13,30	188,00	185,33	170,33	51,00	9,67	73,67	184,84	179,17	181,22	50,98	11,65	70,26
26/01/2015	1121MF	4,50	184,00	180,33	167,00	47,00	9,00	72,00	180,17	175,50	177,11	47,06	10,69	68,82
27/01/2015	1122MF	5,70	177,67	174,67	160,33	49,33	10,00	69,67	174,29	169,00	170,89	49,61	10,08	66,27
28/01/2015	1123MF	6,40	177,33	172,67	156,00	47,00	12,00	69,67	172,48	166,67	168,67	46,88	12,08	65,36
29/01/2015	1124MF	5,10	193,33	191,33	179,33	51,00	7,00	76,00	190,91	186,33	188,00	51,43	10,19	73,07
30/01/2015	1125QMF	1,10	214,33	211,33	202,33	45,00	6,00	84,00	211,33	208,33	209,33	45,00	12,86	81,70
31/01/2015	1126MF	2,20	211,00	208,67	201,00	46,00	5,00	82,67	208,62	206,00	206,89	46,00	10,21	80,78
01/02/2015	1127MF	5,00	198,00	195,33	186,33	46,33	6,00	77,67	195,26	192,17	193,22	46,36	9,28	75,36
02/02/2015	1128MF	3,70	199,00	198,00	188,67	54,33	5,33	77,67	197,56	193,83	195,22	54,36	8,45	76,01
03/02/2015	1129MF	3,00	210,33	207,33	199,33	43,67	5,33	82,33	207,40	204,83	205,67	43,55	10,95	80,33
04/02/2015	1130QMF	2,30	207,67	205,67	196,67	50,00	5,33	81,67	205,46	202,17	203,33	50,00	10,39	79,28
05/02/2015	1131MF	3,80	204,67	202,67	193,67	49,33	5,00	80,33	202,46	199,17	200,33	49,09	9,86	78,10
06/02/2015	1132MF	3,80	199,33	196,00	186,67	44,33	6,67	78,00	196,05	193,00	194,00	44,23	10,22	75,69
07/02/2015	1133MF	5,20	187,33	185,00	172,33	50,67	8,00	73,67	184,60	179,83	181,56	50,67	9,98	70,52
08/02/2015	1134MF	8,50	172,33	169,33	153,67	50,67	10,67	67,33	168,87	163,00	165,11	50,35	10,15	63,92
09/02/2015	1135QMF	11,90	165,33	161,33	142,33	50,00	13,67	65,00	160,84	153,83	156,33	49,63	11,37	60,33
10/02/2015	1136MF	12,60	173,67	171,33	157,67	51,33	9,33	68,33	170,87	165,67	167,56	51,30	8,95	64,97
11/02/2015	1137MF	14,00	160,00	155,67	136,67	49,33	14,33	63,00	155,25	148,33	150,78	48,88	10,94	58,17
12/02/2015	1138MF	23,80	150,67	145,33	121,33	49,33	19,33	59,00	144,77	136,00	139,11	49,10	12,33	53,33
13/02/2015	1139MF	10,80	182,67	180,00	167,67	49,33	8,33	71,33	179,70	175,17	176,78	49,39	9,40	68,69
14/02/2015	1140QMF	10,90	202,00	199,67	190,67	47,67	5,33	79,00	199,53	196,33	197,44	47,73	9,66	76,99
15/02/2015	1141MF	3,00	210,00	208,33	200,00	50,00	5,00	82,33	208,10	205,00	206,11	50,00	10,00	80,39
16/02/2015	1142MF	5,70	193,67	191,67	182,00	50,00	6,33	76,00	191,41	187,83	189,11	49,93	8,68	73,66
17/02/2015	1143MF	2,30	210,00	207,00	199,33	43,33	5,00	82,00	207,09	204,67	205,44	43,09	10,59	80,26
18/02/2015	1144MF	4,90	185,00	182,67	171,33	49,33	7,67	73,00	182,36	178,17	179,67	49,67	8,89	69,87
19/02/2015	1145MF	16,20	171,67	168,33	153,67	49,00	10,33	67,00	168,01	162,67	164,56	48,89	9,75	63,79

20/02/2015	1146MF	12,60	165,00	163,00	146,33	53,67	11,33	64,67	162,25	155,67	158,11	53,57	9,40	61,05
21/02/2015	1147MF	8,90	186,67	184,67	172,67	51,00	7,33	73,00	184,25	179,67	181,33	51,43	9,29	70,46
22/02/2015	1148MF	4,70	199,67	198,33	190,33	52,67	4,67	78,00	198,05	195,00	196,11	52,73	7,81	76,47
23/02/2015	1149MF	4,70	201,33	200,00	192,00	52,33	4,33	79,00	199,72	196,67	197,78	52,32	8,00	77,12
24/02/2015	1150MF	6,10	199,00	198,33	189,33	56,33	5,00	78,00	197,84	194,17	195,56	56,36	7,95	76,14
25/02/2015	1151MF	5,60	200,67	200,67	192,00	60,00	4,33	78,67	200,06	196,33	197,78	60,00	7,38	76,99
26/02/2015	1152MF	7,10	192,67	190,67	181,67	49,00	5,67	75,33	190,46	187,17	188,33	49,03	8,11	73,40
27/02/2015	1153MF	3,50	194,67	193,33	185,00	52,67	5,00	76,33	193,03	189,83	191,00	52,78	7,44	74,44
28/02/2015	1154MF	5,00	189,33	187,33	175,00	51,33	7,33	74,67	186,89	182,17	183,89	51,62	9,84	71,44
01/03/2015	1155MF	4,50	196,33	194,67	185,33	51,33	5,67	77,00	194,36	190,83	192,11	51,18	8,58	74,84
02/03/2015	1156MF	6,20	193,00	191,67	182,00	53,33	5,67	75,67	191,27	187,50	188,89	53,18	8,15	73,53
03/03/2015	1157MF	12,60	180,00	177,33	165,00	49,33	8,00	71,00	177,03	172,50	174,11	49,33	9,09	67,65
04/03/2015	1158MF	5,10	190,33	188,00	178,67	48,00	6,33	75,00	187,84	184,50	185,67	48,11	8,27	72,35
05/03/2015	1159MF	6,00	189,00	187,33	176,33	52,33	6,67	74,33	186,91	182,67	184,22	52,18	8,76	71,63
06/03/2015	1160MF	8,60	171,00	168,00	152,33	50,33	11,00	67,00	167,53	161,67	163,78	50,31	10,00	63,40
07/03/2015	1161MF	8,70	154,33	151,00	129,33	52,00	16,33	60,33	150,18	141,83	144,89	52,03	11,05	55,62
08/03/2015	1162MF	8,80	165,67	162,33	144,67	50,33	12,67	65,00	161,80	155,17	157,56	50,51	10,52	60,85
09/03/2015	1163MF	10,80	167,67	164,00	147,67	49,33	12,00	65,67	163,63	157,67	159,78	49,08	10,27	61,83
10/03/2015	1164MF	11,50	169,67	165,33	147,33	48,33	13,33	66,67	164,98	158,50	160,78	48,34	11,57	62,16
11/03/2015	1165MF	10,80	168,33	165,00	148,00	50,33	12,00	66,00	164,51	158,17	160,44	50,11	10,49	62,03
12/03/2015	1166MF	24,60	169,67	163,33	136,67	48,33	19,33	66,67	162,80	153,17	156,56	48,49	16,19	60,07
13/03/2015	1167MF	47,30	152,00	139,33	92,67	47,33	39,33	59,67	138,73	122,33	128,00	47,19	24,25	47,97
14/03/2015	1168MF	6,40	193,33	191,33	180,33	50,67	6,67	75,67	190,98	186,83	188,33	50,73	9,54	73,27
15/03/2015	1169MF	6,80	187,00	184,33	172,33	49,00	8,00	73,33	184,05	179,67	181,22	49,05	9,74	70,46
16/03/2015	1170MF	9,50	180,33	177,67	165,00	49,67	8,33	71,00	177,34	172,67	174,33	49,67	9,31	67,71
17/03/2015	1171MF	12,10	163,67	160,67	144,33	51,00	12,00	64,00	160,15	154,00	156,22	50,68	9,57	60,39
18/03/2015	1172MF	7,70	181,00	179,33	170,00	51,00	6,33	71,00	179,03	175,50	176,78	51,03	6,92	68,82
19/03/2015	1173MF	8,60	186,00	184,00	172,33	51,00	7,33	73,00	183,60	179,17	180,78	51,21	9,01	70,26
20/03/2015	1174MF	10,50	173,67	171,33	158,67	50,67	9,00	68,33	170,94	166,17	167,89	50,67	8,44	65,16
21/03/2015	1175MF	5,00	190,33	187,67	178,33	46,67	6,00	74,67	187,57	184,33	185,44	46,67	8,49	72,29
22/03/2015	1176MF	4,40	197,00	194,00	185,33	44,67	6,00	77,00	194,02	191,17	192,11	44,55	9,14	74,97
23/03/2015	1177MF	8,40	190,67	188,67	179,67	49,33	5,67	75,00	188,46	185,17	186,33	49,36	7,87	72,61

24/03/2015	1178MF	3,40	202,00	199,67	190,33	48,33	5,67	79,33	199,50	196,17	197,33	48,18	9,92	76,93
25/03/2015	1179MF	2,40	207,00	205,67	196,33	52,67	5,00	81,00	205,29	201,67	203,00	52,73	10,02	79,08
26/03/2015	1180MF	4,20	188,67	187,67	177,33	55,00	6,00	74,33	187,15	183,00	184,56	54,70	7,87	71,76
27/03/2015	1181MF	2,30	198,67	196,00	187,00	46,33	6,00	78,00	195,93	192,83	193,89	46,36	9,39	75,62
28/03/2015	1182MF	4,70	188,67	187,33	176,00	53,67	6,67	74,00	186,82	182,33	184,00	53,79	8,71	71,50
29/03/2015	1183MF	5,10	185,00	181,67	168,67	47,67	9,00	72,67	181,46	176,83	178,44	47,72	10,45	69,35
30/03/2015	1184MF	5,30	189,67	187,67	177,00	50,33	6,67	74,67	187,34	183,33	184,78	50,38	8,84	71,90
31/03/2015	1185MF	7,60	183,33	179,67	166,67	46,67	9,00	72,00	179,53	175,00	176,56	46,76	10,42	68,63
01/04/2015	1186MF	6,70	155,33	150,67	131,67	48,33	15,00	60,67	150,32	143,50	145,89	48,15	10,61	56,27
02/04/2015	1187MF	9,80	161,00	157,67	141,33	50,00	12,00	63,33	157,22	151,17	153,33	49,84	9,47	59,28
03/04/2015	1188MF	7,50	168,00	165,00	150,67	49,67	10,67	65,67	164,63	159,33	161,22	49,58	9,06	62,48
04/04/2015	1189MF	8,00	153,67	148,67	129,67	48,00	16,00	60,00	148,39	141,67	144,00	47,50	10,59	55,56
05/04/2015	1190MF	8,10	135,67	129,33	108,67	46,00	20,00	53,33	129,22	122,17	124,56	45,93	11,05	47,91
06/04/2015	1191MF	10,50	180,67	178,00	168,00	47,33	7,00	71,00	177,86	174,33	175,56	47,46	7,85	68,37
07/04/2015	1192MF	1,50	185,33	183,33	171,33	51,00	8,00	73,00	182,91	178,33	180,00	51,43	9,13	69,93
08/04/2015	1193MF	15,20	186,67	185,33	175,00	53,33	6,00	73,00	184,89	180,83	182,33	53,18	7,86	70,92
09/04/2015	1194MF	7,80	179,67	177,00	167,67	46,67	6,67	70,67	176,91	173,67	174,78	46,75	7,38	68,10
10/04/2015	1195MF	9,70	176,33	174,00	164,00	48,67	6,67	69,00	173,79	170,17	171,44	48,67	7,27	66,73
11/04/2015	1196MF	6,40	194,33	191,67	182,67	46,33	6,00	76,00	191,60	188,50	189,56	46,36	8,77	73,92
12/04/2015	1197MF	21,80	174,00	170,33	153,00	49,67	12,33	68,33	169,89	163,50	165,78	49,55	11,48	64,12
13/04/2015	1198MF	29,50	167,33	162,67	139,67	49,67	16,67	65,33	162,04	153,50	156,56	49,87	13,63	60,20
14/04/2015	1199MF	41,10	172,33	165,33	135,33	48,67	21,33	67,33	164,70	153,83	157,67	48,63	18,29	60,33
15/04/2015	1200MF	20,40	182,33	177,67	160,67	47,33	12,00	71,67	177,46	171,50	173,56	47,20	12,99	67,25
16/04/2015	1201MF	8,00	195,00	193,00	183,67	49,67	5,67	76,67	192,77	189,33	190,56	49,67	8,64	74,25
17/04/2015	1202MF	15,50	183,33	180,67	167,67	50,00	8,67	72,00	180,32	175,50	177,22	49,75	9,85	68,82
18/04/2015	1203MF	16,60	208,67	206,00	197,67	45,67	5,00	82,00	205,98	203,17	204,11	45,45	10,61	79,67
19/04/2015	1204MF	4,10	205,00	203,67	194,67	52,33	5,00	80,33	203,32	199,83	201,11	52,18	9,38	78,37
20/04/2015	1205MF	7,10	193,33	190,67	182,67	45,33	5,67	75,67	190,67	188,00	188,89	45,09	7,96	73,73
21/04/2015	1206MF	12,30	180,00	177,67	165,33	50,33	8,00	70,67	177,29	172,67	174,33	50,48	8,91	67,71
22/04/2015	1207MF	12,50	188,67	186,33	176,00	49,00	6,67	74,00	186,10	182,33	183,67	48,85	8,71	71,50
23/04/2015	1208MF	11,10	193,00	191,33	182,33	51,33	5,67	75,33	191,05	187,67	188,89	51,36	7,93	73,59
24/04/2015	1209MF	10,60	191,33	190,33	180,67	54,67	5,67	75,00	189,87	186,00	187,44	54,55	7,73	72,94

25/04/2015	1210MF	5,90	205,33	204,00	196,00	51,67	4,33	80,33	203,72	200,67	201,78	51,78	8,59	78,69
26/04/2015	1211MF	3,70	213,00	210,33	201,67	46,00	5,33	83,67	210,29	207,33	208,33	45,91	11,91	81,31
27/04/2015	1212MF	4,00	209,33	207,67	200,33	49,67	4,33	82,00	207,50	204,83	205,78	49,56	8,96	80,33
28/04/2015	1213MF	5,90	199,67	197,67	189,33	48,67	5,67	78,00	197,50	194,50	195,56	48,69	8,53	76,27
29/04/2015	1214MF	6,50	201,33	198,67	190,33	45,67	5,00	79,00	198,64	195,83	196,78	45,45	9,30	76,80
30/04/2015	1215MF	7,1	198,00	195,67	186,67	47,67	5,67	78,00	195,53	192,33	193,44	47,67	9,04	75,42
01/05/2015	1216MF	8,8	195,00	194,33	185,67	56,33	5,00	76,33	193,87	190,33	191,67	56,36	7,22	74,64
02/05/2015	1217MF	8,9	196,33	196,33	187,67	60,00	4,67	77,00	195,73	192,00	193,44	60,00	6,88	75,29
03/05/2015	1218MF	10,7	186,67	184,67	176,00	48,67	5,67	73,33	184,48	181,33	182,44	48,73	7,24	71,11
04/05/2015	1219MF	15,2	179,67	176,67	164,67	48,00	8,33	70,67	176,46	172,17	173,67	48,14	9,05	67,52
05/05/2015	1220MF	8,9	208,00	206,33	198,00	50,33	4,67	81,67	206,10	203,00	204,11	50,36	9,64	79,61
06/05/2015	1221MF	7,3	203,00	200,33	192,33	45,00	5,00	79,67	200,33	197,67	198,56	44,91	9,30	77,52
07/05/2015	1222MF	10,9	189,33	186,67	176,67	47,33	6,67	74,33	186,53	183,00	184,22	47,31	8,79	71,76
09/05/2015	1224MF	13	196,33	196,00	187,33	57,67	4,67	77,00	195,46	191,83	193,22	57,78	7,12	75,23
10/05/2015	1225MF	17,3	180,00	176,67	165,33	46,33	8,33	70,67	176,57	172,67	174,00	46,25	8,90	67,71
11/05/2015	1226MF	18,8	178,67	176,00	161,00	51,00	10,00	70,00	175,51	169,83	171,89	50,98	10,38	66,60
12/05/2015	1227MF	23	174,00	170,33	153,33	49,33	12,00	68,33	169,91	163,67	165,89	49,38	11,32	64,18
13/05/2015	1228MF	32	170,67	165,67	144,33	48,67	15,67	67,00	165,22	157,50	160,22	48,59	13,50	61,76
14/05/2015	1229MF	16,1	184,33	181,00	168,67	47,33	8,67	72,33	180,84	176,50	178,00	47,25	9,98	69,22
15/05/2015	1230MF	21,3	197,33	195,00	186,33	47,67	5,67	77,33	194,88	191,83	192,89	47,55	8,70	75,23
16/05/2015	1231MF	7,3	199,00	198,67	189,67	58,00	5,00	78,00	198,11	194,33	195,78	58,00	7,69	76,21
17/05/2015	1232MF	7,8	196,00	195,67	186,00	58,33	5,33	76,67	195,06	191,00	192,56	58,18	7,81	74,90
18/05/2015	1233MF	12,8	187,33	185,33	173,67	51,00	7,00	73,33	184,94	180,50	182,11	51,21	9,17	70,78
19/05/2015	1234MF	24,6	190,67	188,33	176,33	50,00	7,33	74,67	187,98	183,50	185,11	50,29	10,03	71,96
20/05/2015	1235MF	8,2	198,67	197,67	188,33	54,33	5,33	77,67	197,22	193,50	194,89	54,36	8,41	75,88
21/05/2015	1236MF	7,9	197,00	195,67	186,33	52,67	5,67	77,00	195,29	191,67	193,00	52,73	8,43	75,16
22/05/2015	1237MF	9,9	191,00	189,00	179,33	50,00	6,00	75,00	188,74	185,17	186,44	49,85	8,36	72,61
24/05/2015	1239MF	21,4	192,00	189,33	180,00	46,67	6,00	75,00	189,24	186,00	187,11	46,67	8,70	72,94
25/05/2015	1240MF	11	190,00	187,67	177,00	49,00	6,67	74,67	187,41	183,50	184,89	49,19	9,10	71,96
26/05/2015	1241MF	8,3	196,00	195,67	186,33	58,33	5,33	76,67	195,08	191,17	192,67	58,18	7,57	74,97
27/05/2015	1242MF	10	188,67	188,67	179,67	60,00	5,00	74,33	188,04	184,17	185,67	60,00	6,35	72,22
28/05/2015	1243MF	13,9	183,33	179,67	168,00	45,67	8,33	72,00	179,62	175,67	177,00	45,67	9,66	68,89

29/05/2015	1244MF	16,9	182,67	178,33	164,33	45,67	10,00	71,67	178,26	173,50	175,11	45,85	11,26	68,04
30/05/2015	1245MF	10,1	192,33	190,00	179,67	48,67	6,33	75,33	189,77	186,00	187,33	48,81	9,17	72,94
31/05/2015	1246MF	9,5	192,00	189,00	179,00	46,00	6,67	75,33	188,93	185,50	186,67	46,10	9,35	72,75
01/06/2015	1247MF	9,7	192,00	189,33	180,33	46,33	6,00	75,00	189,26	186,17	187,22	46,36	8,47	73,01
02/06/2015	1248MF	11,1	189,33	187,67	177,33	51,67	6,33	74,33	187,29	183,33	184,78	51,62	8,37	71,90
03/06/2015	1249MF	16,2	182,67	179,33	166,00	48,00	9,00	71,67	179,10	174,33	176,00	47,96	10,33	68,37
04/06/2015	1250MF	16,4	177,00	174,00	159,33	49,67	10,00	69,00	173,60	168,17	170,11	49,80	10,17	65,95
05/06/2015	1251MF	16,9	181,67	178,33	165,67	47,67	9,00	71,00	178,15	173,67	175,22	47,50	9,84	68,10
06/06/2015	1252MF	16,2	181,33	178,00	164,33	48,00	9,33	71,00	177,74	172,83	174,56	48,14	10,34	67,78
07/06/2015	1253MF	24,5	178,67	174,00	154,33	48,67	13,33	70,00	173,60	166,50	169,00	48,50	13,75	65,29
08/06/2015	1254MF	14,9	183,00	179,67	166,33	48,00	9,00	71,67	179,43	174,67	176,33	48,01	10,38	68,50
09/06/2015	1255MF	16,7	182,00	178,67	165,33	48,33	9,33	71,33	178,43	173,67	175,33	48,06	10,25	68,10
10/06/2015	1256MF	14,7	184,67	181,67	169,33	48,33	8,33	72,67	181,43	177,00	178,56	48,33	9,83	69,41
11/06/2015	1257MF	23,2	187,33	184,67	172,33	49,33	8,00	73,67	184,36	179,83	181,44	49,39	9,97	70,52
12/06/2015	1258MF	11	192,67	190,33	182,00	47,00	5,67	75,67	190,24	187,33	188,33	46,91	7,88	73,46
13/06/2015	1259MF	6,3	205,33	204,33	195,00	54,33	5,00	80,33	203,89	200,17	201,56	54,18	9,42	78,50
14/06/2015	1260MF	5	196,33	194,00	185,67	47,00	5,67	77,00	193,91	191,00	192,00	46,91	8,33	74,90
15/06/2015	1261MF	3,9	208,33	207,00	198,00	53,00	4,67	81,67	206,65	203,17	204,44	52,73	9,99	79,67
16/06/2015	1262MF	54,3	203,00	202,00	192,67	54,33	5,00	79,67	201,56	197,83	199,22	54,44	9,04	77,58
17/06/2015	1263MF	5,7	199,00	197,67	189,33	51,67	5,00	78,00	197,36	194,17	195,33	51,78	7,95	76,14
18/06/2015	1264MF	68,3	182,33	180,67	170,00	52,00	7,00	71,67	180,27	176,17	177,67	52,05	7,82	69,08
19/06/2015	1265MF	53,1	177,67	176,00	163,67	52,67	8,00	69,33	175,49	170,67	172,44	52,86	8,30	66,93
20/06/2015	1266MF	7,3	190,67	189,00	179,33	51,33	6,00	75,00	188,67	185,00	186,33	51,21	8,10	72,55
21/06/2015	1267MF	14,1	181,00	178,00	165,33	48,67	8,67	71,00	177,74	173,17	174,78	48,50	9,57	67,91
22/06/2015	1268MF	19,9	176,00	172,67	157,00	49,67	10,67	69,00	172,27	166,50	168,56	49,51	10,73	65,29
23/06/2015	1269MF	14,4	189,00	187,33	177,33	51,67	6,00	74,33	186,98	183,17	184,56	51,67	8,12	71,83
24/06/2015	1270MF	7,7	198,67	195,67	187,67	44,00	6,00	78,00	195,74	193,17	194,00	43,64	8,90	75,75
25/06/2015	1271MF	13,1	184,00	181,33	169,33	49,00	8,00	72,33	181,05	176,67	178,22	49,14	9,36	69,28
26/06/2015	1272MF	16,8	177,00	174,00	157,33	51,00	11,00	69,33	173,46	167,17	169,44	50,84	11,20	65,56
27/06/2015	1273MF	17,9	169,00	166,00	148,00	51,33	12,67	66,33	165,37	158,50	161,00	51,42	10,88	62,16
28/06/2015	1274MF	18,1	174,67	171,67	154,00	51,00	11,67	68,67	171,06	164,33	166,78	51,29	11,40	64,44
29/06/2015	1275MF	28,9	170,67	165,67	144,00	48,67	15,67	67,00	165,20	157,33	160,11	48,75	13,65	61,70

30/06/2015	1276MF	26,9	168,00	164,33	143,67	51,00	14,33	65,67	163,66	155,83	158,67	51,05	12,27	61,11
01/07/2015	1277MF	19,9	178,00	175,33	161,67	50,33	9,00	69,67	174,94	169,83	171,67	50,25	9,59	66,60
02/07/2015	1278MF	6,5	203,00	201,67	192,00	53,00	5,00	80,00	201,27	197,50	198,89	52,73	9,57	77,45
03/07/2015	1279MF	12,3	193,00	191,00	179,00	51,00	7,00	75,67	190,58	186,00	187,67	51,43	10,15	72,94
04/07/2015	1280MF	13,9	191,33	188,67	177,33	48,33	7,00	75,00	188,43	184,33	185,78	48,57	9,91	72,29
05/07/2015	1281MF	15,4	187,33	183,67	169,00	48,00	10,00	73,33	183,41	178,17	180,00	48,07	11,93	69,87
06/07/2015	1282MF	23,7	182,33	177,33	158,33	48,00	13,00	71,33	177,05	170,33	172,67	47,50	14,17	66,80
07/07/2015	1283MF	16,9	187,67	183,33	168,67	46,33	10,33	74,00	183,22	178,17	179,89	46,40	12,38	69,87
08/07/2015	1284MF	17,1	171,33	167,33	148,33	50,00	13,33	67,00	166,84	159,83	162,33	49,63	12,08	62,68
09/07/2015	1285MF	16,6	178,67	174,33	158,00	47,67	11,33	70,00	174,10	168,33	170,33	47,55	11,93	66,01
10/07/2015	1286MF	22,1	159,00	156,33	136,00	53,00	14,00	62,00	155,47	147,50	150,44	53,04	10,70	57,84
11/07/2015	1287MF	14,6	178,67	175,33	161,67	48,33	9,33	70,00	175,08	170,17	171,89	48,21	10,02	66,73
12/07/2015	1288MF	11	188,67	186,33	175,00	49,33	7,00	74,33	186,03	181,83	183,33	49,67	9,34	71,31
13/07/2015	1289MF	18,2	173,00	169,67	152,67	50,33	12,00	67,67	169,18	162,83	165,11	50,19	11,03	63,86
14/07/2015	1290MF	20,6	174,33	171,33	151,67	52,33	13,33	68,67	170,59	163,00	165,78	52,12	12,32	63,92
15/07/2015	1291MF	32	170,00	164,00	137,00	49,00	19,33	66,67	163,37	153,50	157,00	49,08	16,26	60,20
16/07/2015	1292MF	27,4	175,00	171,33	152,00	50,67	13,00	68,33	170,75	163,50	166,11	50,43	12,57	64,12
17/07/2015	1293MF	23,7	168,67	164,00	139,67	50,33	17,33	66,00	163,28	154,17	157,44	50,36	14,38	60,46
18/07/2015	1294MF	13,3	178,00	173,67	157,67	47,67	11,33	69,67	173,46	167,83	169,78	47,39	11,67	65,82
19/07/2015	1295MF	7,2	191,33	188,67	179,33	46,67	6,33	75,00	188,57	185,33	186,44	46,75	8,61	72,68
20/07/2015	1296MF	8,4	192,33	190,33	180,33	50,33	6,33	75,33	190,05	186,33	187,67	50,10	8,74	73,07
21/07/2015	1297MF	12,5	193,67	191,33	181,00	49,00	6,67	76,00	191,10	187,33	188,67	48,85	9,36	73,46
22/07/2015	1298MF	12,7	177,33	174,33	160,33	49,33	9,67	69,33	173,98	168,83	170,67	49,39	9,86	66,21
23/07/2015	1299MF	14,7	172,00	168,67	152,00	50,00	12,00	67,33	168,20	162,00	164,22	50,00	10,75	63,53
24/07/2015	1300MF	9,7	188,00	186,00	174,67	50,67	6,67	73,67	185,63	181,33	182,89	50,71	9,04	71,11
25/07/2015	1301MF	9,9	185,67	182,67	171,00	47,67	8,00	73,00	182,48	178,33	179,78	47,81	9,57	69,93
26/07/2015	1302MF	11,5	186,67	184,33	172,33	50,00	8,00	73,33	183,98	179,50	181,11	50,29	9,50	70,39
27/07/2015	1303MF	9,4	186,00	183,33	171,00	49,33	8,00	73,00	183,03	178,50	180,11	49,33	9,80	70,00
28/07/2015	1304MF	16,4	134,67	128,67	100,67	49,33	25,00	53,00	127,97	117,67	121,33	49,39	14,45	46,14
29/07/2015	1305MF	9,8	186,67	184,67	172,67	51,00	7,67	73,33	184,25	179,67	181,33	51,43	9,29	70,46
30/07/2015	1306MF	6,8	190,67	189,67	179,67	55,00	6,00	75,00	189,18	185,17	186,67	54,55	7,88	72,61
31/07/2015	1307MF	6,1	204,33	202,33	193,00	49,67	5,33	80,00	202,10	198,67	199,89	49,55	10,06	77,91

01/08/2015	1308MF	10,8	184,00	181,33	169,00	49,33	8,00	72,33	181,03	176,50	178,11	49,33	9,56	69,22
02/08/2015	1309MF	18,1	185,33	182,33	170,00	48,33	8,33	73,00	182,10	177,67	179,22	48,33	9,91	69,67
03/08/2015	1310MF	20,8	174,33	171,67	151,67	52,67	13,00	68,33	170,83	163,00	165,89	52,82	12,31	63,92
04/08/2015	1311MF	16,3	160,67	157,00	136,00	51,00	15,67	63,00	156,30	148,33	151,22	51,10	11,56	58,17
05/08/2015	1312MF	15,7	166,00	163,00	145,00	51,33	12,67	65,00	162,37	155,50	158,00	51,42	10,55	60,98
06/08/2015	1313MF	10	191,33	189,00	178,33	49,00	6,67	75,00	188,74	184,83	186,22	49,07	9,26	72,48
07/08/2015	1314MF	33,6	64,33	65,00	50,33	62,67	22,67	25,00	63,83	57,67	59,89	62,76	12,72	22,61
08/08/2015	1315MF	16,8	159,00	154,00	127,00	50,67	20,00	62,00	153,16	143,00	146,67	50,62	14,28	56,08
09/08/2015	1316MF	27,6	160,67	154,67	121,33	51,00	24,33	63,00	153,59	141,00	145,56	50,84	17,25	55,29
10/08/2015	1317MF	43,3	160,67	151,00	109,00	48,33	32,33	63,00	150,09	134,83	140,22	48,78	21,50	52,88
11/08/2015	1318MF	25,1	167,00	162,00	137,33	50,00	17,67	45,33	161,32	152,17	155,44	49,88	14,42	59,67
12/08/2015	1319MF	13,2	188,33	186,33	174,33	51,00	7,00	74,00	185,91	181,33	183,00	51,43	9,50	71,11
13/08/2015	1320MF	8,9	197,67	196,33	185,33	53,67	6,33	77,67	195,84	191,50	193,11	53,57	9,71	75,10
15/08/2015	1322MF	7,1	201,33	199,67	189,67	51,00	5,33	79,00	199,32	195,50	196,89	51,19	9,80	76,67
16/08/2015	1323MF	6,4	203,67	202,67	192,00	54,67	5,33	80,00	202,13	197,83	199,44	54,55	10,19	77,58
17/08/2015	1324MF	6,2	197,00	194,67	185,67	47,67	6,00	77,33	194,53	191,33	192,44	47,73	8,90	75,03
18/08/2015	1325MF	12,3	181,00	178,67	166,00	50,67	8,33	71,00	178,27	173,50	175,22	50,64	9,20	68,04
19/08/2015	1326MF	13,1	187,00	185,00	171,67	51,67	8,33	73,33	184,49	179,33	181,22	52,06	10,13	70,33
20/08/2015	1327MF	20,4	177,00	173,00	155,00	49,33	12,33	69,33	172,58	166,00	168,33	49,17	12,36	65,10
21/08/2015	1328MF	26,9	174,33	169,33	146,67	49,33	16,33	68,33	168,80	160,50	163,44	49,14	14,64	62,94
22/08/2015	1329MF	14,7	186,00	183,67	170,67	51,00	8,33	73,00	183,25	178,33	180,11	50,83	10,00	69,93
23/08/2015	1330MF	7,6	198,67	196,33	186,33	48,67	6,33	78,00	196,12	192,50	193,78	48,59	9,86	75,49
24/08/2015	1331MF	9,8	209,67	207,33	199,67	46,33	4,67	82,33	207,29	204,67	205,56	46,32	9,94	80,26
25/08/2015	1332MF	10,7	192,67	190,33	180,33	48,33	6,33	75,33	190,12	186,50	187,78	48,51	8,99	73,14
26/08/2015	1333MF	15,6	193,00	190,67	180,33	48,67	6,33	75,67	190,43	186,67	188,00	48,81	9,27	73,20
27/08/2015	1334MF	8,3	202,33	200,67	190,67	51,67	5,67	79,33	200,32	196,50	197,89	51,52	9,97	77,06
28/08/2015	1335MF	12,5	193,33	191,33	180,67	50,33	6,33	76,00	191,01	187,00	188,44	50,48	9,31	73,33
29/08/2015	1336MF	32	177,00	171,00	147,00	48,00	17,00	69,33	170,58	162,00	165,00	48,00	16,13	63,53
30/08/2015	1337MF	35,7	173,33	166,33	138,67	48,00	20,33	68,00	165,87	156,00	159,44	47,88	17,51	61,18
31/08/2015	1338MF	19,6	187,00	182,67	166,00	47,67	11,33	73,67	182,41	176,50	178,56	47,51	13,37	69,22
01/09/2015	1339MF	14	192,33	190,00	179,00	49,33	6,67	75,33	189,72	185,67	187,11	49,52	9,61	72,81
02/09/2015	1340MF	17,6	183,33	179,33	166,00	46,00	9,33	72,00	179,24	174,67	176,22	46,08	10,79	68,50

03/09/2015	1341MF	15,6	182,33	180,00	167,67	50,33	8,33	71,33	179,63	175,00	176,67	50,54	9,16	68,63
04/09/2015	1342MF	19,2	176,67	174,00	160,67	50,00	9,00	69,00	173,63	168,67	170,44	49,90	9,26	66,14
05/09/2015	1343MF	11,6	178,00	175,67	162,67	51,00	8,67	69,67	175,25	170,33	172,11	50,89	9,06	66,80
06/09/2015	1344MF	18,6	179,67	177,00	163,00	50,33	9,33	70,67	176,58	171,33	173,22	50,37	9,96	67,19
07/09/2015	1345MF	7,5	189,33	187,67	177,33	51,67	6,33	74,33	187,29	183,33	184,78	51,64	8,37	71,90
08/09/2015	1346MF	11	179,67	176,00	163,00	46,67	9,00	70,67	175,86	171,33	172,89	46,76	9,96	67,19
09/09/2015	1347MF	13,6	175,33	172,33	158,67	49,67	9,33	69,00	172,01	167,00	168,78	49,31	9,47	65,49
10/09/2015	1348MF	6,2	205,33	204,00	194,67	52,67	5,00	80,33	203,63	200,00	201,33	52,73	9,71	78,43
11/09/2015	1349MF	13,6	174,00	171,67	159,33	50,33	8,67	68,00	171,29	166,67	168,33	50,38	8,30	65,36
12/09/2015	1350MF	9,7	196,67	193,67	185,00	44,67	6,00	77,33	193,69	190,83	191,78	44,55	9,09	74,84
13/09/2015	1351MF	8,2	208,67	207,67	198,33	54,67	5,00	82,00	207,22	203,50	204,89	54,55	10,05	79,80
14/09/2015	1352MF	6,6	206,67	205,00	196,33	50,33	5,00	81,00	204,74	201,50	202,67	50,36	9,66	79,02
15/09/2015	1353MF	11,6	198,67	199,33	190,33	63,67	4,67	78,00	198,56	194,83	196,11	63,64	7,48	76,41
16/09/2015	1354MF	6,5	208,00	207,67	200,33	57,00	3,67	81,67	207,22	204,17	205,33	57,14	7,54	80,07
17/09/2015	1355MF	4,9	211,33	209,33	203,00	45,67	3,67	82,67	209,31	207,17	207,89	45,40	8,70	81,24
18/09/2015	1356MF	5,3	186,33	183,67	174,67	46,33	6,00	73,00	183,60	180,50	181,56	46,36	7,83	70,78
19/09/2015	1357MF	9,3	174,67	172,33	159,33	51,00	9,00	68,67	171,91	167,00	168,78	50,92	8,71	65,49
20/09/2015	1358MF	11,4	176,33	174,33	161,00	52,33	9,00	69,00	173,82	168,67	170,56	52,17	8,88	66,14
21/09/2015	1359MF	10,7	184,00	181,67	169,00	50,67	8,00	72,33	181,27	176,50	178,22	50,67	9,56	69,22
22/09/2015	1360MF	10,5	184,33	183,00	171,33	54,00	7,33	72,67	182,46	177,83	179,56	54,07	8,66	69,80
23/09/2015	1361MF	10,4	177,33	174,33	160,00	49,67	9,67	69,67	173,96	168,67	170,56	49,61	10,04	66,14
24/09/2015	1362MF	7,8	183,67	181,00	170,00	48,00	7,67	72,00	180,79	176,83	178,22	48,24	8,74	69,35
25/09/2015	1363MF	25,3	169,00	166,00	148,33	51,00	12,00	66,00	165,39	158,67	161,11	51,29	10,73	62,22
26/09/2015	1364MF	12,9	171,67	169,67	153,00	54,00	11,00	67,33	168,92	162,33	164,78	53,68	10,08	63,66
27/09/2015	1365MF	12,2	181,33	178,33	165,00	49,00	9,00	71,00	178,03	173,17	174,89	48,97	9,98	67,91
28/09/2015	1366MF	10	182,00	180,00	167,33	51,67	8,00	71,33	179,53	174,67	176,44	51,81	9,13	68,50
29/09/2015	1367MF	9,7	181,67	179,00	166,67	49,33	8,00	71,33	178,70	174,17	175,78	49,33	9,28	68,30
30/09/2015	1368MF	8,2	187,33	187,33	175,67	59,67	6,33	73,67	186,52	181,50	183,44	59,74	8,43	71,31
01/10/2015	1369MF	10,6	189,00	186,33	175,33	48,33	7,33	74,33	186,12	182,17	183,56	48,31	9,38	71,44
02/10/2015	1370MF	11,6	182,33	180,67	168,33	52,67	8,00	71,33	180,15	175,33	177,11	52,86	8,79	68,76
03/10/2015	1371MF	14,2	172,00	168,67	152,67	49,67	11,33	67,33	168,25	162,33	164,44	49,63	10,43	63,66
04/10/2015	1372MF	13,5	175,00	171,00	155,33	48,00	11,00	68,33	170,74	165,17	167,11	47,84	10,95	64,77

05/10/2015	1373MF	10,7	203,67	203,00	193,33	56,33	5,00	79,67	202,46	198,50	200,00	56,18	9,15	77,84
06/10/2015	1374MF	6,5	209,67	209,33	201,00	57,67	4,00	82,00	208,82	205,33	206,67	57,78	8,72	80,52
07/10/2015	1375MF	4,9	201,00	200,33	190,33	55,67	5,67	79,00	199,77	195,67	197,22	55,52	9,90	76,93
08/10/2015	1376MF	7,4	181,33	180,00	168,33	54,00	7,67	71,33	179,46	174,83	176,56	53,96	8,56	68,69
09/10/2015	1377MF	10	175,00	173,67	160,67	54,33	8,33	68,67	173,04	167,83	169,78	54,48	8,43	65,88
10/10/2015	1378MF	12,6	167,33	165,67	149,33	54,33	11,33	66,00	164,87	158,33	160,78	54,39	9,50	62,16
11/10/2015	1379MF	4,1	198,33	197,33	187,67	54,33	5,67	78,00	196,87	193,00	194,44	54,55	8,90	75,75
12/10/2015	1380MF	3,2	210,67	209,67	200,67	54,00	5,00	82,33	209,25	205,67	207,00	54,00	10,14	80,65
22/10/2015	1390MF	14,5	167,00	164,33	147,33	52,00	11,67	65,33	163,70	157,17	159,56	51,84	10,05	61,63
23/10/2015	1391MF	10,1	167,33	164,67	150,00	50,67	10,33	65,33	164,20	158,67	160,67	50,78	9,00	62,22
24/10/2015	1392MF	13,7	138,33	134,00	112,00	50,00	19,00	54,33	133,37	125,17	128,11	50,14	10,52	49,08
25/10/2015	1393MF	14,1	153,33	150,33	130,00	52,33	15,00	60,00	149,54	141,67	144,56	52,19	10,29	55,56
26/10/2015	1394MF	10	161,00	159,33	143,33	54,33	11,00	63,33	158,56	152,17	154,56	54,44	8,77	59,74
27/10/2015	1395MF	4,4	208,33	207,67	198,67	56,00	4,67	81,67	207,18	203,50	204,89	56,00	9,38	79,80
28/10/2015	1396MF	8,6	206,00	204,00	195,67	48,67	4,67	81,00	203,84	200,83	201,89	48,69	9,56	78,76
29/10/2015	1397MF	16,4	196,00	194,00	184,67	49,67	6,00	76,67	193,77	190,33	191,56	49,55	8,77	74,64
30/10/2015	1398MF	15,1	162,00	160,67	145,33	55,33	10,33	63,67	159,87	153,67	156,00	55,15	8,22	60,26
31/10/2015	1399MF	14	174,00	172,00	159,67	51,33	8,33	68,00	171,56	166,83	168,56	51,62	8,13	65,42
01/11/2015	1400MF	11,1	187,00	186,00	176,33	54,67	5,33	73,33	185,53	181,67	183,11	54,67	7,28	71,24
02/11/2015	1401MF	6,9	203,33	202,33	192,67	55,00	5,33	79,67	201,87	198,00	199,44	55,00	9,37	77,65
03/11/2015	1402MF	25,6	196,33	194,67	186,33	51,00	5,33	77,00	194,43	191,33	192,44	50,91	7,87	75,03
04/11/2015	1403MF	5,9	188,00	188,00	180,00	60,00	4,00	73,67	187,44	184,00	185,33	60,00	5,63	72,16
05/11/2015	1404MF	12,2	199,33	198,00	189,33	52,33	5,33	78,00	197,67	194,33	195,56	52,22	8,24	76,21
06/11/2015	1405MF	11,4	177,33	175,33	163,67	51,00	7,67	69,33	174,94	170,50	172,11	51,21	8,09	66,86
07/11/2015	1406MF	18,1	128,33	124,67	104,67	50,67	18,33	50,33	124,04	116,50	119,22	50,78	10,16	45,69
08/11/2015	1407MF	17	157,00	154,00	138,00	50,67	12,00	61,67	153,51	147,50	149,67	50,51	8,83	57,84
09/11/2015	1408MF	14,7	151,67	148,00	130,67	49,67	14,00	59,67	147,56	141,17	143,44	49,51	9,22	55,36
10/11/2015	1409MF	18,9	142,67	138,33	118,33	49,33	17,33	56,00	137,84	130,50	133,11	49,33	9,77	51,18
11/11/2015	1410MF	18,6	127,00	122,33	103,00	48,67	19,00	49,67	121,96	115,00	117,44	48,33	10,44	45,10
12/11/2015	1411MF	32,4	107,67	103,33	85,00	48,67	21,00	42,00	102,96	96,33	98,67	48,56	11,77	37,78
13/11/2015	1412MF	53	111,33	107,67	90,33	49,33	18,67	43,67	107,22	100,83	103,11	49,60	10,41	39,54
14/11/2015	1413MF	52,2	130,33	124,67	101,67	48,00	22,33	51,00	124,25	116,00	118,89	48,10	12,36	45,49

15/11/2015	1414MF	30,6	147,67	143,33	123,00	49,33	16,67	58,00	142,82	135,33	138,00	49,49	10,31	53,07
16/11/2015	1415MF	15,1	159,00	155,33	139,00	49,33	12,67	62,00	154,96	149,00	151,11	49,08	9,43	58,43
17/11/2015	1416MF	8,9	162,33	159,67	145,33	50,67	10,33	64,00	159,22	153,83	155,78	50,64	8,40	60,33
18/11/2015	1417MF	22	150,33	147,33	129,67	51,33	13,67	58,67	146,73	140,00	142,44	51,27	8,99	54,90
19/11/2015	1418MF	43,6	135,67	133,00	113,33	53,00	16,33	53,00	132,18	124,50	127,33	52,81	8,97	48,82
20/11/2015	1419MF	11	151,00	147,67	127,00	51,67	16,00	59,33	146,92	139,00	141,89	51,67	10,35	54,51
21/11/2015	1420MF	14,7	186,33	182,67	166,00	49,33	11,00	73,00	182,27	176,17	178,33	49,19	12,90	69,08
22/11/2015	1421MF	4,9	201,67	199,67	189,67	49,67	6,00	79,00	199,39	195,67	197,00	49,55	10,11	76,73
23/11/2015	1422MF	5,3	189,67	188,00	176,00	52,33	7,00	74,33	187,51	182,83	184,56	52,75	9,47	71,70
24/11/2015	1423MF	4,8	186,67	184,67	172,00	51,67	8,00	73,33	184,20	179,33	181,11	51,81	9,69	70,33
25/11/2015	1424MF	6,1	191,67	189,67	177,00	51,33	7,33	75,00	189,20	184,33	186,11	51,61	10,37	72,29
26/11/2015	1425MF	9,1	184,67	182,33	169,00	51,00	8,67	72,33	181,89	176,83	178,67	51,14	10,02	69,35
27/11/2015	1426MF	6,1	186,33	183,67	172,00	48,67	7,67	73,00	183,41	179,17	180,67	48,95	9,45	70,26
28/11/2015	1427MF	5,8	180,33	177,00	163,00	48,33	9,33	70,67	176,72	171,67	173,44	48,50	10,40	67,32
29/11/2015	1428MF	6,7	169,67	166,33	149,67	50,33	12,00	66,67	165,87	159,67	161,89	50,03	10,48	62,61
30/11/2015	1429MF	12,6	133,00	128,33	103,33	50,33	22,33	52,00	127,56	118,17	121,56	50,51	12,56	46,34
01/12/2015	1430MF	15,3	117,33	111,33	88,00	48,00	25,00	45,67	110,96	102,67	105,56	47,75	14,29	40,26
02/12/2015	1431MF	18,1	108,67	103,00	81,00	47,67	25,33	42,33	102,65	94,83	97,56	47,70	14,59	37,19
03/12/2015	1432MF	20,6	95,33	91,00	74,00	48,00	22,33	37,67	90,72	84,67	86,78	47,89	12,60	33,20
04/12/2015	1433MF	26,3	71,33	71,33	59,00	60,33	18,00	28,33	70,47	65,17	67,22	60,37	9,92	25,69
05/12/2015	1434MF	23,9	76,33	74,67	60,00	54,00	21,33	30,00	73,99	68,17	70,33	54,08	11,98	26,73
06/12/2015	1435MF	17,8	104,67	98,67	78,67	46,33	25,00	41,00	98,53	91,67	94,00	46,14	14,19	35,95
07/12/2015	1436MF	20,5	111,67	106,33	81,67	49,33	27,00	44,00	105,73	96,67	99,89	49,30	15,52	37,91
08/12/2015	1437MF	22,1	113,33	108,00	84,67	49,00	25,33	44,33	107,49	99,00	102,00	48,83	14,48	38,82
09/12/2015	1438MF	14,8	112,67	108,00	85,67	49,67	24,00	44,00	107,42	99,17	102,11	49,65	13,62	38,89
10/12/2015	1439MF	16,6	134,67	129,33	107,33	48,33	20,00	53,00	128,91	121,00	123,78	48,26	11,30	47,45
11/12/2015	1440MF	14	136,00	130,33	110,00	46,67	19,00	53,00	130,10	123,00	125,44	46,92	10,57	48,24
12/12/2015	1441MF	13,5	148,67	143,67	122,00	48,67	18,00	58,00	143,20	135,33	138,11	48,72	11,14	53,07
13/12/2015	1442MF	14,5	142,67	138,00	115,33	49,67	19,00	56,00	137,39	129,00	132,00	49,69	10,84	50,59
14/12/2015	1443MF	18	163,00	158,00	136,67	48,67	16,33	64,00	157,56	149,83	152,56	48,59	12,52	58,76
15/12/2015	1444MF	24,3	171,00	165,00	138,67	49,00	19,00	67,00	164,42	154,83	158,22	48,86	16,14	60,72
19/12/2015	1445MF	30,1	86,67	81,33	62,33	46,67	28,33	34,00	81,12	74,50	76,78	46,80	16,34	29,22

20/12/2015	1446MF	24,8	99,67	93,67	69,33	48,00	30,67	39,00	93,22	84,50	87,56	48,16	17,95	33,14
21/12/2015	1447MF	5,6	163,33	161,67	147,67	53,67	9,33	64,00	161,04	155,50	157,56	53,65	7,87	60,98
22/12/2015	1448MF	12,7	124,33	119,00	99,00	47,33	20,33	49,00	118,72	111,67	114,11	47,38	11,34	43,79
23/12/2015	1449MF	13,9	111,33	106,67	86,67	48,67	22,00	43,67	106,25	99,00	101,56	48,63	12,46	38,82
24/12/2015	1450MF	10,4	129,00	124,67	103,67	49,67	19,67	50,67	124,11	116,33	119,11	49,70	10,90	45,62
25/12/2015	1451MF	24	102,33	98,67	80,33	50,00	21,67	40,33	98,15	91,33	93,78	50,00	12,05	35,82
26/12/2015	1452MF	27,9	108,00	103,33	84,00	48,67	22,33	42,67	102,96	96,00	98,44	48,36	12,50	37,65
27/12/2015	1453MF	19	121,67	116,33	93,00	48,67	23,33	47,67	115,82	107,33	110,33	48,78	13,36	42,09
28/12/2015	1454MF	10,8	162,67	159,67	142,00	51,33	12,67	63,67	159,06	152,33	154,78	51,10	10,05	59,74
29/12/2015	1455MF	5,5	204,33	202,67	192,67	51,33	5,67	80,00	202,32	198,50	199,89	51,51	10,31	77,84
30/12/2015	1456MF	4,8	189,33	188,33	177,33	55,00	6,33	74,33	187,77	183,33	185,00	55,03	8,38	71,90
31/12/2015	1457MF	4,5	205,33	203,00	194,00	48,00	5,33	80,33	202,86	199,67	200,78	47,93	10,24	78,30

Saharan Dust Outbreaks days are identified in orange color.

© 2019 by the authors. Submitted for possible open access publication under the terms and conditions of the Creative Commons Attribution (CC BY) license (http://creativecommons.org/licenses/by/4.0/).
